# Evaluating the acceptability, feasibility, and implementation fidelity of a multipurpose mobile health (mHealth) app used for Tuberculosis (TB) contact tracing by Ward-Based Outreach Teams (WBOTs) in South Africa

**DOI:** 10.1371/journal.pgph.0007120

**Published:** 2026-08-31

**Authors:** Don Lawrence Mudzengi, Lindiwe Tsope, Piotr Hippner, Fezeka Mboniswa, Thapelo Mpanza, Tanyaradzwa Dube, Richard Lessells, Indira Govender, Dumile Gumede, Alison D. Grant, Katherine Fielding, Candice M. Chetty-Makkan, Kavindhran Velen, Salome Charalambous

**Affiliations:** 1 The Aurum Institute, Johannesburg, South Africa; 2 University of the Witwatersrand, School of Public Health, Johannesburg, South Africa; 3 Africa Health Research Institute, School of Laboratory Medicine & Medical Sciences, College of Health Sciences, University of KwaZulu-Natal , Durban, South Africa; 4 TB Centre, London School of Hygiene and Tropical Medicine, London, United Kingdom; 5 Durban University of Technology, Faculty of Health Sciences, Durban, South Africa; 6 Health Economics and Epidemiology Research Office (HE²RO), Johannesburg, South Africa; PLOS: Public Library of Science, UNITED STATES OF AMERICA

## Abstract

Mobile health (mHealth) technologies are increasingly used to support community-based healthcare. However, their real-world impact often remains unclear. Understanding implementation factors is essential for advancing their use and achieving meaningful health outcomes. We evaluated an mHealth tool (AitaHealth) after customising its modules and workflows for household Tuberculosis (TB) contact tracing and other community-based data collection by community health workers (CHWs). We describe the acceptability, feasibility, and implementation fidelity of this approach. We conducted a mixed-methods evaluation in two South African districts: uMkhanyakude and Ekurhuleni. We collected qualitative data through focus group discussions (FGDs) and in-depth interviews (IDIs) with CHWs, team leaders, and key stakeholders. We used deductive thematic analysis grounded in the Technology Acceptance Model (TAM) to assess the acceptability and implementation feasibility of the mHealth tool. We used quantitative data from the AitaHealth metadata to assess implementation fidelity. CHWs appreciated AitaHealth’s efficiency, data security, and credibility. Across the two districts, 103 CHWs recorded data for 2,452 households and 10,649 household members. However, they reported challenges in ease of use, with unreliable devices, weak support, and safety concerns hindering data collection. These issues led to inconsistent engagement, with 48.5% of CHWs logging in fewer than 15 times during implementation. Despite these challenges, when used, AitaHealth ensured high-quality data collection and household coverage, with TB-related fields completed in over 94% of households, demonstrating its potential under better conditions. AitaHealth`s limitations stemmed from system constraints rather than user resistance. To achieve full impact, mHealth tools require reliable infrastructure and supportive environments for both the tools and their implementers.

## Introduction

Tuberculosis (TB) remains a global health concern. In 2023, 10.8 million people developed TB, 1.25 million died, and 4.2 million cases went undiagnosed; the WHO thus continues to recommend targeted contact tracing [1]. In South Africa, an estimated 270,000 people developed TB in 2023 [1]. However, around 20% were never reached by testing or treatment services, resulting in missed diagnoses and downstream inefficiencies, including poor linkage to care and fewer opportunities for TB preventive treatment (TPT) among household contacts [2–5].

In 2011, South Africa introduced ward-based outreach teams (WBOTs) to strengthen community-based primary healthcare [6]. These teams consist of Community Health Workers (CHWs) and their team leaders, who provide a broad spectrum of community-based services, including TB contact tracing and other related services. However, they frequently encounter delays, data loss, and missed follow-ups due to their reliance on paper-based systems [7–10], as well as limited resources and competing demands [6,8,11–14]. Although mobile health (mHealth) tools like Mobenzi and CommCare can ease routine tasks by enabling quicker data capturing, automated reminders, defined workflows and real-time monitoring and reporting [7–9,15–17], their impact on TB outcomes remains unclear [18,19], and integration into the health system is limited [20]. A South African TB Think Tank review noted that most tools had not been formally evaluated, making it challenging to justify their wider adoption [21]. Decisions about scale-up rely not only on effectiveness but also on implementation outcomes such as feasibility, acceptability, and fidelity [22]. A trial in Uganda illustrated this; it was only through a detailed process evaluation that researchers were able to understand why the mHealth strategy failed to increase the proportion of household contacts who completed TB evaluation within 14 days, and why it did not improve case detection compared to standard clinic-based contact investigation [23,24]. These findings highlight the importance of implementing science evaluations.

We conducted a mixed-methods implementation science study, the Asibambisane project, to evaluate an mHealth approach delivered by CHWs using AitaHealth, a multipurpose digital platform. While AitaHealth was routinely used to support data collection across multiple health and social areas, we tailored it for TB contact tracing for this study. This study evaluates the tool’s acceptability, feasibility, and implementation fidelity in real-world outreach conditions. Although AitaHealth was used in this study, the evaluation aimed to generate insights relevant to broader mHealth use in TB contact tracing. Specifically, we used the mixed-methods approach to answer the following questions: (i) was the platform acceptable to CHWs, team leaders, and healthcare workers? (ii) was it feasible for CHWs to use during routine contact tracing? and (iii) was itimplemented as intended in real-world outreach?

## Materials and methods

### Study Background and Setting

We conducted the Asibambisane study from 2017 to 2022 to improve TB contact tracing using a multipurpose mHealth tool in uMkhanyakude (KwaZulu-Natal), two districts with high TB and HIV burdens but contrasting settings.

uMkhanyakude is a predominantly rural area with approximately 625,000 inhabitants over an area of 12,818 km^2^ and a population density of 49 people per km^2^ [25]. Scattered traditional homesteads make household visits time-consuming for CHWs, who walk long distances. uMkhanyakude faces high poverty and unemployment levels and a significant disease burden [26]. In 2022, uMkhanyakude had the highest antenatal HIV prevalence in the country (44%) [27]. In uMkhanyakude, TB was the second-leading cause of death after HIV, according to mortality data from 2012–2017 as reported in the 2023/24 Integrated Development Plan and District Health Barometer [25,28].

In contrast, Ekurhuleni is an urban district with over 3 million residents spanning 1,975 km^2^ (approximately 1,159 people per km^2^) [29,30]. It includes townships and informal settlements, and, despite being more economically developed than uMkhanyakude, still faces considerable poverty [29,30]. In 2022, it had the highest antenatal HIV prevalence in Gauteng province (28.5%) [27].

We selected these two districts because they offered contrasting implementation contexts: one rural and one urban, and because our teams were already active in both. Their high TB and HIV burdens, along with well-established CHW outreach systems, made them ideal for testing an mHealth tool under real-world conditions.

### The AitaHealth application

AitaHealth is an mHealth tool designed by the Family Medicine Department at the University of Pretoria to support CHWs with household-level data collection and health service delivery. It was developed in partnership with Mezzanine Ware [31]. It supports data collection and monitoring across multiple health areas, including maternal and child health, chronic illness, HIV, TB, and social determinants of health [31,32]. AitaHealth is easily customisable and can be modified to collect other data such as sanitation, water access, food security, common illness symptoms, pregnancy status, and chronic conditions [33]. Supervisors can access this data via a secure web portal for real-time oversight, planning, and reporting. AitaHealth also supports offline “store-and-forward” functionality for areas with low connectivity [31].

AitaHealth was a general-purpose data-collection platform with no disease-specific focus. It allowed users to customise modules to programme needs. For our study, we worked with the developers to strengthen the TB contact-tracing questions and workflows, enabling Department of Health staff to complete household contact tracing more consistently. We retained other routine household-care modules, such as maternal health, because the same staff also delivered broader primary care services during household visits. Thus, the intervention focused on optimising the TB contact-tracing component within a broader household data-collection platform. We provided each CHW with a new commercially available device: the Vodafone Tab Mini 7 tablet, preloaded with AitaHealth; the tablets ran on Android version 6.0 software for the AitaHealth application [34]. The tablet used a MediaTek 1.3 GHz quad-core Central Processing Unit (CPU), a Mali-400 MP2 Graphics Processing Unit (GPU), had 1GB Random Access Memory (RAM), and 8GB of storage, expandable via microSD up to 32 GB [34]. It was powered by a 2580 mAh lithium-ion (Li-ion) battery and featured a 7-inch thin-film transistor (TFT) display with a resolution of 600 x 1024 pixels, as well as a built-in global positioning system (GPS). AitaHealth is designed for Android devices and is not compatible with Apple’s iPhone operating system (iOS).

### Study outreach procedures

**Fig 1:**
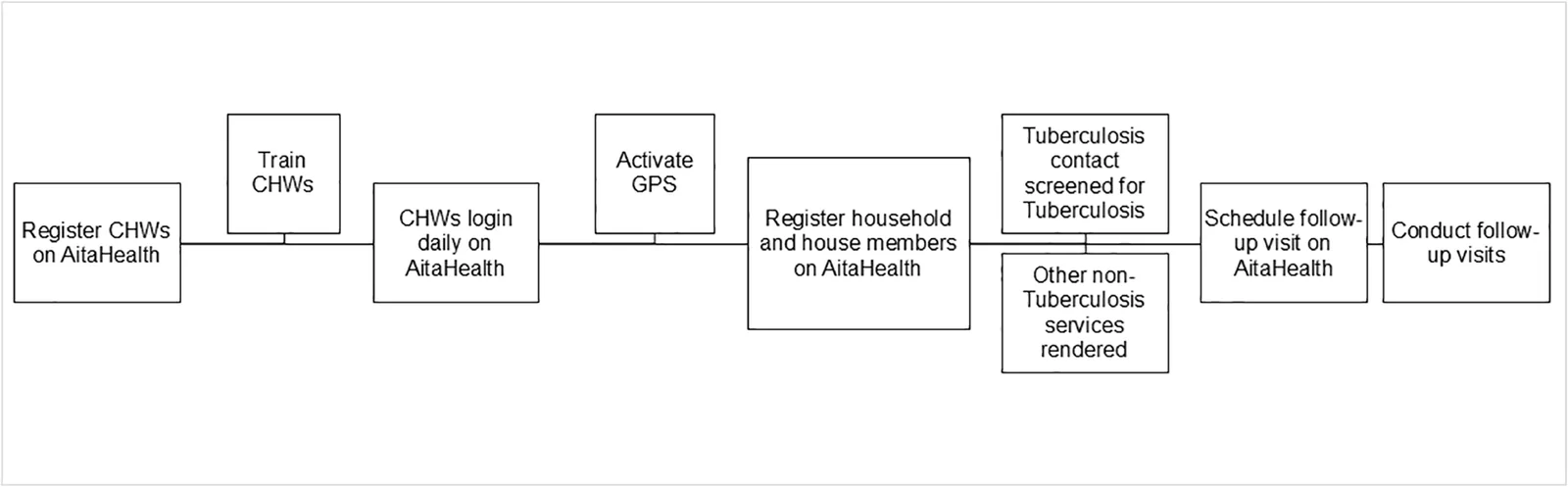
Basic AitaHealth Process Flow.

Before we introduced AitaHealth in uMkhanyakude and Ekurhuleni, CHWs in both districts had been using paper-based tools to routinely collect household-level health and socioeconomic data as part of their day-to-day WBOT responsibilities. Their team leaders compiled this information into monthly reports for monitoring and evaluation; however, the manual process was time-consuming and often resulted in incomplete or inaccurate records. AitaHealth had also been widely used by CHWs in the Tshwane District (Gauteng Province, South Africa) to digitise similar workflows [31,32]. We drew on this experience and introduced AitaHealth to replace paper tools in uMkhanyakude and Ekurhuleni. Fig 1 illustrates how CHWs in uMkhanyakude and Ekurhuleni used AitaHealth during the study period, mirroring the standard WBOT data collection cycle by adding TB-specific screening and follow-up tasks tailored for this evaluation. The study team designed and deployed the digital tool, customised the AitaHealth platform, trained CHWs, and provided technical support during rollout. District teams managed routine operations, logistics, supervision, and incident management.

Before implementation, CHWs in uMkhanyakude and Ekurhuleni received a once-off, in-person AitaHealth training session in their respective districts delivered by the developers. Training was standardised across sites and conducted shortly before rollout. It covered how to use AitaHealth, including navigation, entering household-level data, and operating it during outreach. To assess competency, CHWs completed practical tasks of capturing dummy data directly into AitaHealth during the session. It also included basic TB management, contact tracing, and TB preventive treatment, led by facilitators from the University of Pretoria’s Department of Family Medicine. Although no formal refresher training was provided during the implementation period for using AitaHealth, CHWs received ongoing remote technical support as needed.

### Data collection

As part of the Asibambisane study, CHWs in uMkhanyakude used AitaHealth to collect data from June 2018 to March 2019. In Ekurhuleni, data collection using AitaHealth began in September 2018 and continued until September 2019. After September 2019, the Ekurhuleni district adopted AitaHealth and continued its use, eventually transitioning to the CommCare app in 2021. CommCare retained the same functionalities developed during the study. We define active days as days when AitaHealth was accessible to CHWs and outreach days as days when CHWs recorded data using the platform.

We developed interview guides using the Technology Acceptance Model, which guided our coding and analysis (see S1 File and S2 File). The Technology Acceptance Model helped assess acceptability by exploring how intuitive CHWs found AitaHealth (ease of use) and how well it supported daily tasks (usefulness). We adopted feasibility and fidelity definitions from Proctor, Silmere (22), defining feasibility as the extent to which CHWs could use AitaHealth under real-world conditions and fidelity as the degree to which CHWs used the tool as intended.

We collected qualitative and quantitative data to assess AitaHealth’s acceptability, feasibility, and fidelity. We conducted focus group discussions (FGDs) with CHWs and in-depth interviews (IDIs) with CHW team leaders, TB nurses, and key stakeholders, including district quality improvement officers and programme managers. In uMkhanyakude, we conducted all IDIs and FGDs in November 2018, at the peak of the implementation. In Ekurhuleni, data collection was more spread out; we conducted interviews and FGDs between March 2019 and May 2022. We conducted the final interviews in Ekurhuleni after the CHWs had already transitioned to a CommCare-based mHealth tool similar to AitaHealth.

We used convenience sampling for FGDs, with team leaders assisting in identifying available CHWS without disrupting field activities. For IDIs, we included all available team leaders, nurses, and outreach stakeholders from an exhaustive list of potential participants. We monitored responses during data collection and stopped when no new themes emerged, indicating that saturation had been reached.

Trained research assistants, fluent in English, isiZulu, and other local languages, used semi-structured interview guides for FGDs and IDIs to collect qualitative data in participants’ preferred languages. Each FGD included 12 participants and lasted 90–120 minutes, while IDIs lasted 45–60 minutes. We obtained written informed consent and recorded all sessions with permission.

### Ethical considerations

The study received ethical approval in South Africa from the University of the Witwatersrand Human Research Ethics Committee (reference number 170505) on 11 October 2017, with a five-year validity period ending on 10 October 2022. Additional approval was granted by the London School of Hygiene & Tropical Medicine (LSHTM) Ethics Committee (reference number 11020) on 11 August 2017.

CHWs registered the first household using AitaHealth on 18 June 2018 in uMkhanyakude, and the final household was registered on 02 September 2019 in Ekurhuleni. Focus group discussions (FGDs) and in-depth interviews (IDIs) were conducted in uMkhanyakude on 22–23 November 2018 and in Ekurhuleni between 29 March 2019 and 13 May 2022.

All FGD and IDI participants were employees or stakeholders from the Department of Health. Participation was voluntary, and all individuals provided written informed consent in their preferred language. No minors were included in the study.

### Data analysis

We analysed qualitative data thematically using a deductive approach guided by the Technology Acceptance Model. We transcribed all FGDs and IDIs verbatim and translated them into English where necessary. The first author quality-checked transcripts to verify that the transcribed text matched the audio recording. Five coders independently analysed a subset of transcripts from both sites and data types. After resolving discrepancies through discussion, we finalised a coding framework based on the Technology Acceptance Model components: perceived usefulness, ease of use, attitudes, and facilitating conditions.

We analysed quantitative data using Stata version 16 (Stata Corp, College Station, TX, USA). We prepared selected tables in Microsoft Excel, whilst coding qualitative data with a combination of Quirkos software (Quirkos Ltd, Edinburgh, UK) and manual coding to support thematic analysis.

We analysed quantitative data using descriptive statistics, including proportions, means, medians, and frequencies, to summarise CHW engagement, system usage, and data completeness. Table 1 outlines the indicators and data sources used for each outcome.

**Table 1:** List of indicators for AitaHealth process evaluation.

Indicator/ AitaHealth Process Step	Data Collection		Data Analysis
	Quantitative	Qualitative	
**Fidelity: Did AitaHealth function as planned?**			
**CHWs trained on AitaHealth/ Train CHWs.** *Tracks if all CHWs received the planned training before implementation.*	Training logs, attendance records	CHW feedback on training effectiveness	% trained, completion rates
**CHWs registered on AitaHealth/ Register CHWs** *Assesses if all trained CHWs were successfully on-boarded to AitaHealth.*	System registration logs	CHW-reported registration challenges	% or # registered CHWs
**AitaHealth use on outreach days/ CHWs login daily** *Measures how often AitaHealth was accessed for data collection.*	Login timestamps, system access logs	CHW feedback on use patterns	% outreach days used, trends
**User engagement/ CHWs login daily** *Evaluate how consistently CHWs interacted with the system.*	Login frequency	CHW-reported engagement barriers	% of CHWs logging in, trends
**Households recorded/ Register households.** *Tracks the number of households entered into AitaHealth.*	Household registration logs	CHW reflections on the registration process	Counts, means, medians
**Triaging of household members/ Register households** *Monitors screening for Tuberculosis and other conditions at a household level.*	Triage records in AitaHealth	CHW experiences with the triage process	% households triaged; average number of members screened
**Number of outcomes recorded/ Tuberculosis screening & non-Tuberculosis services** *Documents services provided, including Tuberculosis screenings and referrals.*	Service records on AitaHealth	CHW reflections on service documentation	% of recorded health outcomes per visit
**Feasibility: Were conditions supportive for implementation?**			
**System Factors (AitaHealth software functionality)** *Examines whether AitaHealth functioned reliably without frequent errors.*		CHW reports on system failures	Deductive thematic analysis using qualitative data collected in FGDs and IDIs
**Hardware Factors (CHW device performance)** *Assesses whether tablets functioned properly without major disruptions.*		CHW reports on battery and performance issues	
**Infrastructure Factors (Network & connectivity)** *Measures the impact of connectivity on data syncing and access.*		CHW-reported network failures	
**Technical Support (Availability of IT troubleshooting)** *Tracks the effectiveness of support provided when CHWs encounter issues.*		CHW feedback on resolution times	
**Human Factors (CHW knowledge & skills)/ Train CHWs** *Evaluate whether CHWs had the required skills to use AitaHealth effectively.*		CHW-reported usability challenges	
**External Barriers (Environmental & logistical constraints)** *Identifies challenges outside the system, such as travel difficulties and infrastructure.*		CHW-reported challenges	
**Acceptability: Did CHWs perceive AitaHealth as useful and usable?**			
**Ease of use:** *Examines whether CHWs found the system easy to navigate and use.*		CHW perceptions of system usability	Deductive thematic analysis using qualitative data collected in FGDs and IDIs
**Usefulness** *Assess whether AitaHealth improved CHW workflow and efficiency.*		CHW perceptions on workflow impact	
**Barriers & facilitators/ All steps** *Identifies key factors influencing AitaHealth adoption.*		CHW surveys, focus groups	

### Definition of Terms

**Active days:** The total number of days AitaHealth was made available for use by CHWs in the field.

**Outreach days:** The number of days on which CHWs actually used AitaHealth to record data. Outreach days are a subset of active days.

**Triaging:** The completion of screening questions by CHWs during household visits.

## Results

### Qualitative Results: Acceptability and Feasibility

We conducted four FGDs in total, with two done in each district (Ekurhuleni and uMkhanyakude). Each FGD had 12 participants, for a total of 48 across the 4 FGDs in the two districts. Each district contributed two IDIs with TB nurses and two with CHW team leaders; Ekurhuleni had two additional IDIs with key stakeholders. (Table 2).

**Table 2:** Summary of qualitative data collection.

Category	Ekurhuleni	uMkhanyakude	Total
Data collection period	2019–2022	2021	
Age of FGD participants *(years),* mean; p50, (±sd)	41.8; 37.5 (±9.4)	44.7; 45, (±7.4)	43.3; 42.5, (8.9)
Years of service	*10.3; 11 (±0.9)	*7.6; 7, (±4.4)	8.9; 10, (3.4)
**Focus Group Discussions (FGDs)**	**2**	**2**	**4**
- ^1^*CHWs in FGDs*	*24*	*24*	*48*
**In-Depth Interviews (IDIs) (Total)**	**6**	**4**	**10**
*- Tuberculosis Nurses in IDIs*	*2*	*2*	*4*
*- CHW Team Leaders in IDIs*	*2*	*2*	*4*
*- Key Stakeholders in IDIs*	*2*	*0*	*2*

1 *CHWs -Community Health Workers*

* p ≤ 0.05

*Data were collected in uMkhanyakude (2021) and in Ekurhuleni (IDIs 2019–2021; FGDs 2022)*

For uMkhanyakude, all data were collected over two consecutive days in November 2018 (22–23 November). In Ekurhuleni, IDIs were conducted from March 2019 to September 2021, with FGDs held in May 2022. Demographic information was collected only for FGD participants. The median age was 45 years in uMkhanyakude and 37.5 years in Ekurhuleni. Using Wilcoxon rank-sum tests appropriate for these unpaired groups, age differences were not statistically significant (exact p = 0.11). By contrast, years of service differed significantly: CHWs in Ekurhuleni had longer service histories (median, 11 vs 7 years), as confirmed by a rank-sum test (exact p = 0.005) (Table 3).

**Table 3:** Summary of feasibility findings, implications and recommendations.

Theme	Key Issues from Results	Implications for Feasibility	Recommended Actions
Battery life	Short battery life disrupted CHW work; overheating and shutdowns.	Unreliable battery performance disrupts tool usability in daily practice.	Invest in quality devices with longer battery life; including power backups or solar chargers.
Hardware failures	Devices freezing and failing to charge; aging equipment led to reverting to paper.	Without quality devices and replacement cycles, digital tools fail under real-world pressure.	Budget for regular device replacement; match the hardware to workload demands.
Software malfunctions	Registration bugs: data loss when households had many members.	Technical malfunctions reduce trust and drive users back to paper-based work.	Fix bugs promptly; build responsive software support channels.
Connectivity challenges	Poor sync due to network instability, even in urban areas.	Even with offline mode, sync delays affect supervision and oversight.	Design for offline use but improve network planning; include sync protocols.
System inefficiencies (technical support)	No real-time tech support; reliance on workarounds; no escalation process.	Support gaps threaten sustainability and introduce governance issues.	Include funded support channels (e.g., help-desks); plan for local capacity building.
Security concerns	CHWs felt unsafe carrying tablets in high-risk areas.	CHW anxiety reduces field usage, threatening sustained adoption.	Create safety protocols, insure devices, and train CHWs on secure use.
Employment precarity	Unclear job security reduced willingness to adopt new tools.	Worker insecurity affects motivation and digital engagement.	Stabilise CHW employment terms; align digital rollout with HR strategy.

### Perceived usefulness of the AitaHealth to CHWs

CHWs reported that AitaHealth improved their data collection efficiency, accuracy, and reliability. It addressed longstanding frustrations with paper-based systems, enhanced data security, and simplified screening and referral processes. Many CHWs also felt that using AitaHealth made their work appear more professional and respected in the community.

### A reliable, efficient way to work

Paper records often got lost or damaged, disrupting follow-ups and patient care. CHWs noted that AitaHealth resolved these issues by keeping information safe and accessible.

“…*the tablets are helpful because all the information is in one place and easy to access; unlike paper, it can get lost, or I get rained on, and I lose all the information.* …” ***KZN FGD 1***

Several CHWs also appreciated the time saved through digital entry, noting that it reduced paperwork and allowed their visits to be more focused.

*“We took too much time when writing on the paper here; it saves our information; its stored correctly with paper can get misplaced, lost, or wet when it rains, but here the information is always updated.”* ***EKN FGD 1***

### Completeness of data collection

CHWs reported that AitaHealth improved the consistency and quality of data captured during visits. Where they previously relied on memory to identify patients needing follow-up, AitaHealth automatically flagged individuals who require referral. AitaHealth also ensured that screening steps were not skipped and that important questions were always asked.

*“…we were using paper; we would skip some screenings; we did not screen everything, so AitaHealth guides you, and you will not skip anything because it tells you what to screen, so with paper, you could skip and not do everything.”* ***EKN FGD 2***

*“Before, we would skip some questions because we did not see their need, but when you use a tablet, it tells you which questions to ask.…., you do not just randomly decide which questions you want to ask when you want.”* ***KZN FGD 1***

### Improving data security and patient confidentiality

CHWs felt that AitaHealth provided a safer and more reliable way to store and manage patient records. Unlike paper, which was often lost, shared, or damaged, AitaHealth kept information protected and easily retrievable.

*“It helps because people’s information is safe, but with AitaHealth, its safe, and it can store so that when I want to follow up, I just go to follow up, when I want tracing, I just go to straight to tracing, when I want to register registration only, so it saves a lot of things.”* ***EKN FGD 2***

### Compiling of statistics

CHWs reported that AitaHealth made compiling statistics easier than paper-based methods.

*“Another thing is that there on AitaHealth that stats are from AitaHealth. It counts all numbers and gather all the work from all the people under the supervisor. It does this automatically. AitaHealth counts on its own, and we just go in and view the stats, all the numbers will be combined.”* ***KZN FGD 2***

Many CHWs appreciated how AitaHealth automated the process of compiling data. Instead of manually counting numbers at the end of the month, they could instantly access consolidated reports across their team.

*“It has helped me a lot with stats. I use to struggle compiling stats using paper …. it has helped me a lot.”* ***EKN FGD 2***

### Proof that they were working

Unlike paper records that could be filled in retrospectively, AitaHealth provided real-time evidence of CHWs’ field activities. Although they showed some scepticism about the need to prove they were working, this gave supervisors clearer visibility into who was working and what had been done.

*“I thank you for the project that has helped us with the tablets because when you are working in the community, it becomes difficult to know who actually works and who does not, but using the tablet shows who in the field is working. ….you can literally see what everyone gets up to.”* ***KZN FGD 1***

GPS tracking further strengthened this, as supervisors could see CHWs’ locations and verify their activities remotely.

*“It is nice to work with AitaHealth because you can see the GPS and the house, and the supervisor can see you, and when you finish, you send. The supervisor can see that this one worked and finished, and this one did not finish.; AitaHealth makes it easy than taking a paper and reading.”* ***KZN FGD 1***

### Respect and credibility

CHWs shared that using AitaHealth changed how they were perceived in their communities. The tablet made their work more visible and structured, reinforcing their role as professionals.

*“I was really happy [laughs]. I told myself I would now use a tablet, thinking that it will really show that I work. It’s better than always writing on paper; people that also see me will see that this thing that I am doing really exists…”* ***KZN FGD 2***

Additionally, stakeholders noted that the tablet gave CHWs more confidence and brought structure to their work.

*“Other than just being taken as just community health workers just going around but now when they had some gadgets it gave them confidence and they were able to do contact tracing and write the feedback on their gadgets and so on” –* ***EKN Key Stakeholder 2***

Community members appeared to take CHWs more seriously when they used AitaHealth, viewing the device as a marker of professionalism.

*“I noticed that by continuing using AitaHealth with this community we might make a lot of progress, and the community might be motivated and inspired and see that this thing that we do is real because using paper they might just think that you are playing. But when they see you with AitaHealth and they say, “I have this and this sickness before you even ask, and they go to the clinic and get treatment.”* ***KZN FGD 2***

### Perceived usefulness of AitaHealth to team leaders, healthcare workers, and stakeholders

AitaHealth provided supervisors and healthcare workers with better oversight of contact tracing activities, enhancing accountability, data accuracy, and follow-up capabilities. The shift from paper-based to digital systems enabled real-time monitoring and simplified reporting, even in areas with poor network coverage.

#### Enhanced real-time supervision and oversight.

Supervisors noted that AitaHealth improved transparency by capturing and displaying CHWs’ locations and activities in real time, reducing the risk of falsified reports and missed follow-ups.

*“…it also shows whether the community health workers are doing their work, because they must record where they are. … and it also for me the benefit also its because it reminds them what to do, and also what questions to ask”.* ***EKN-Key Stakeholder 2***

#### Simplified monitoring and reporting.

AitaHealth made it easier for healthcare workers to track fieldwork without relying on handwritten notes.

*“In the tablet, I can see..even if I am here, I can open and see what time..carer was in the household and how many household members are registered so far. Even if there is a follow-up, I can even check the follow-up”* ***EKN HealthCare Worker 1***

#### Improved data quality and clinic statistics.

Stakeholders reported that AitaHealth improved the completeness and visibility of TB data, enabling more cases to be identified and enhancing reporting.

“…*the numbers yes they have improved, I also believe and think that some of the patients were not reported on the TB register before … but I think all these interventions there were some improvement*.” ***Key Stakeholder (QI Coach) KZN***

The system’s offline capability was especially useful in rural settings, allowing CHWs to continue working uninterrupted.

“*One must not worry about the fact that they are in an area where there is no network cause at the end of the day the information will be uploaded. I was also worried about the network thing because I experienced it in some homes that I visited but when I got to work, I found that the work had been uploaded*.” ***KZN FGD 1***

### Perceived ease of use of AitaHealth

The ease of using AitaHealth was linked to training, experience, and technical challenges. CHWs generally found the system easier to use the more they used it, but gaps in training, issues with older users, and technology failures affected its usability.

### Learning through experience

Many CHWs initially found AitaHealth difficult, but they became more confident in using it with time.

*“the more time you use the tablet. It’s easy to use the tablet and on your spare time one must play within, so they get familiar with it. If one day you use it and another day you, don’t you will forget because you only use it sometimes, it’s easier and you learn faster if you have it and use it daily”* ***KZN FGD 1***

### Impact of inadequate training

While regular use improved CHWs’ confidence, gaps in training created challenges, particularly in follow-ups, which required users to return to households they had previously registered.

“*We were taught how to register someone…. Where the time was short was when they trained us on follow-ups that is when you have to return to the home you registered people, we did not get enough time with that…we were not allocated enough time on follow up and we did not finish, and I was left behind.”* ***KZN FGD 1***.

### Difficulties faced by older staff with technology and lack of immediate support

Older CHWs found the system more challenging to use, requiring additional mentorship and refresher courses to build confidence.

“*we don’t know technology; technology is for small children [giggles]. ….Every now and then we need to do this because we are old and because of that we do not grasp things as quick as kids*.” ***KZN FGD 1***

Some participants also mentioned the lack of immediate support as a factor further frustrating the ease of use of AitaHealth.

*“As number 15 has said the tablet makes it easy to work, it did make things easy, but we used to bother number 20 (a more experienced CHW familiar with technologies and troubleshooting) when we are stuck.”* ***KZN FGD 2***

### Implementation Feasibility

Technological barriers significantly affected AitaHealth’s feasibility. These included battery issues, hardware problems, software malfunctions, and connectivity constraints impacting the efficiency of CHWs using the system.

### Battery life

The tablets’ short battery life was a major challenge that prevented CHWs from completing their tasks. The batteries drained quickly, often shutting down mid-visit, resulting in data loss, delayed reporting, and leading some to return to paper recording.

*“It’s like the battery isn’t strong enough I don’t know what happened but sometimes it just gets hot then switches off. I have no idea what would have happened because you end up not having anywhere to write your information.… sometimes it gets hot then gets blank. Sometimes you find that the information that they had captured disappears”* ***KZN FGD 1***

Due to these limitations, some CHWs expressed a need for higher-quality devices.

*“I cannot be specific, but these are [Name of brand] tabs, There are other brands of high standards that make better tools than the ones we are using.”* ***KZN FGD 2***

In some locations, CHWs had to travel to clinics or health facilities just to charge their devices, wasting valuable time.

*“ there were no electricity they had to come here in and charge them and wait for 1 hour to 2 hours wait for them until they are full so that they can go back to work”* ***EKN HealthCare Worker***

### Hardware limitations

Even when fully charged, many tablets were prone to sudden failures, freezing, and unresponsiveness, making it difficult for CHWs to complete data entry.

*“Also, another thing, AitaHealth`s get tired. You find that you are using it, and it just stops working. It gets stuck because you have been using it continuously for different things. “* ***KZN FGD 2***

Some CHWs mentioned that their devices failed to charge properly, rendering them unusable.

*“Others no longer work like us we no longer have one we are back to paper. We use it but some no longer use it because their gadgets have been damaged, some its charging points, others it is the screen. It just went off”* ***EKN FGD 2***

However, an argument was presented that device failures were primarily due to prolonged use rather than misuse, particularly given that many of the tablets had been in circulation for several years, which may have led many to revert to using paper.

*“So that is why I can say maybe it is because of- maybe it has been two years now so its 2019 until now. yah its already 2 years when they have started using the phones so and most of them the problem when we are saying its broken, they are no longer charging.”* ***EKN Key stakeholder 5***

### Software malfunctions

Beyond hardware issues, software glitches significantly affected CHWs’ ability to complete their tasks.

These software challenges led CHWs to revert to paper-based data collection, thereby reducing the effectiveness of digital tracking.

*“Sometimes I was forced to write down on paper so that all people were registered because if I did not do that, I would not be able to check their illnesses because they would be deleted if I added more people* ***.” KZN FGD 2***

### Connectivity and network limitations

Network challenges were reported in both rural and urban areas, challenging the assumption that connectivity would be more stable in Ekurhuleni. The primary limitation was the inability to sync data in real time, which delayed supervisory oversight rather than disrupting data capture.

*“The other challenge obviously was that network challenge where we are not able unable to sync and send the work to the Team Leader”* ***EKN DOH 1***

However, key stakeholders, CHWs, and team leaders agreed that this did not stop the work from continuing. AitaHealth’s offline functionality ensured uninterrupted data collection, with information syncing once a connection was restored.

*“If there is no network, we would continue to collect data and as soon the network wherever you, are you just sync it will tell exactly where the data collected”* ***EKN Key Stakeholder***

### Security concerns regarding device theft

Some CHWs are worried about using the tablets in the field due to safety concerns. These concerns were not limited to rural settings, as CHWs in urban areas also worried about being targeted while working in the community.

*“But frankly speaking there is no safety for us we are just risking our lives, so we just pray that God protects us when we enter household to be able to work and go back to the clinic, because who is going to help us when we get robbed If it is not the community.”* ***EKN FGD 002***

*“We work in a forest do you understand when we say forest? We pass through these forests with a tablet, and you see working at a place like that is risky because you might just meet someone with a knife and take it (the tool). We do need maybe strong bags, and they can be protect the tool while we cross rivers, we use pathways in the forest, anyone can come my way I need to protect my life that means I have to give him the tool so that I can be safe.”* ***KZN FGD 2***

While team leaders acknowledged the safety concerns, they believed basic precautions, such as being more discreet while working in the field, could mitigate this problem.

*“People are stealing the tablets they must just make sure that they keep the tablets safe they mustn’t leave them unattended. We told them not to show off with the tablets in the streets they must only take them out when they are in the household, but so far, we didn’t hear anything but for safety reasons in the fields this will help.”* ***EKN HealthCare Worker 1***

### Remuneration

CHWs expressed dissatisfaction with the lack of remuneration associated with the study and with being broadly undermined by the health system. Some CHWs had expected the tool to include financial incentives, but when it did not, they concluded that its benefits would accrue only to researchers. They explicitly stated that they stopped using the tool because they did not receive any remuneration, while for some, it led to misconceptions that they could earn more by using the tool.

*“We did have expectations that we would get a bit of money. That is the other reason why people end up stopping to use the tool and do the DOH job because that at least gives them something* ***….****but the person who started it and the owner of the tool benefit from this the most…..The principal investigator.”* ***KZN FGD002***

***“****Even in my family they think that I have a better salary since I am now using a tablet.”* ***(EKN FGD002)***

## Summary of feasibility findings

### Quantitative results: Implementation Fidelity

#### AitaHealth Use on Active days.

All CHWs completed their training before using AitaHealth, achieving a 100% completion rate because it was integrated into their routine tasks and made mandatory. AitaHealth was used on just over half of the weekdays in both sites: 54.4% in Ekurhuleni and 50.2% in uMkhanyakude. Some weekend use was also recorded, with CHWs using the tool on 12.1% of weekends in Ekurhuleni and 11.4% in uMkhanyakude. These figures reflect moderate use of the tool during outreach. Table 4 shows the full usage breakdown, including outreach days and implementation duration.

**Table 4:** Overall AitaHealth usage statistics.

District	Ekurhuleni	uMkhanyakude
**Dates when the study was implemented**		
*Start date*	20-Sep-2018	18-Jun-2018
*End date*	02-Sep-2019	27-Mar-2019
*Months of implementation*	11.5 months	9 months
**Day when AitaHealth was available for use**	**347**	**282**
*Working weekdays*	248	203
*Weekends*	99	79
**Days when AitaHealth was used by CHWs**	**147**	**111**
*Weekday use*	135 (54.4% of weekdays)	102 (50.2% of weekdays)
*Weekend use*	12 (12.1% of weekends)	9 (11.4% of weekends)
*% day with any use*	42.4% (147/347)	39.4% (111/282)
*% of days timestamped use*	38.3% (133/347)	34.8% (98/282)

### CHW Engagement and System Use Patterns

A total of 103 CHWs participated in AitaHealth in uMkhanyakude and Ekurhuleni. During the study, CHWs recorded 2,452 households and 10,649 household members, which were entered into AitaHealth as individual entries (Table 5). Of the individual entries, 83.6% were timestamped, indicating a high proportion of real-time data capture. However, AitaHealth use varied significantly among users and fell short of the expected levels of routine engagement.

**Table 5:** Overall household and data entry summary.

Metric	Ekurhuleni	uMkhanyakude	Total
**A. CHW Deployment**			
*Total CHWs*	61	42	103
**B. Data Captured in AitaHealth**			
*Total entries recorded*	5,096	5,553	10,649
*Entries with timestamps (%)*	4,677 of 5,096 (91.8%)	4,213 of 5,553 (75.9%)	8,890 of 10,649 (83.6%)
*Households recorded*	1,514	938	2,452
*Household size,* mean; p50, (±sd)	4.3; 4, (±2.4)	7.9; 7, (±4.0)	6.2; 6, (±3.8)
*Households per CHW,* mean; p50, (±sd)	24.8; 18, (±25.7)	22.3; 19.5, (±16.4)	23.8; 18.2, (±22.3)
*Timestamped entries per CHW,* mean; p50, (±sd)	76.7; 60, (±68.2)	100.3; 48.5, (±118.7)	86.3; 57.9, (±92.4)
*Timestamped entries per outreach day,* mean; p50, (±sd)	35.2; 26 (±38)	43; 26, (±43.1)	38.5; 26, (±40.3)
**C. CHW Login Activity** *(61 in Ekurhuleni, 42 in uMkhanyakude)*			
*CHWs with 75–136 logins*	5 (8.2%)	5 (11.9%)	10 (9.7%)
*CHWs with 30–74 logins*	10 (16.4%)	8 (19.0%)	18 (17.5%)
*CHWs with 15–29 logins*	15 (24.6%)	10 (23.8%)	25 (24.3%)
*CHWs with 5–14 logins*	16 (26.2%)	12 (28.6%)	28 (27.2%)
*CHWs with 1–4 logins*	15 (24.6%)	7 (16.7%)	22 (21.4%)

Table 5 summarises the household and data entry statistics. CHWs recorded a median of 18 households in Ekurhuleni and 19.5 in uMkhanyakude, with corresponding means of 24.8 (±25.7) and 22.3 (±16.4). The median number of timestamped entries per CHW was 60 in Ekurhuleni and 48.5 in uMkhanyakude. Across the implementation period, AitaHealth was available for use on 347 days in Ekurhuleni and 282 days in uMkhanyakude. CHWs submitted timestamped entries on 133 days (38.3%) in Ekurhuleni and 98 days (34.8%) in uMkhanyakude. On these outreach days, CHWs recorded a median of 26 timestamped entries per day in both districts, with interquartile ranges of 28 in Ekurhuleni and 65 in uMkhanyakude. Login activity was skewed; in Ekurhuleni, just over half (52.1%) logged in at least once, compared to 90.2% in uMkhanyakude. In uMkhanyakude, CHWs recorded 43 timestamped entries per day, with 90.2% recording at least once. These figures indicate partial adherence to daily use, as each CHW was to record at least one entry per active day.

Login activity shows inconsistent use. Half of CHWs (48.5%) logged in fewer than 15 times during implementation, which is less than once a month. Only 10 CHWs (9.7%) had more than 75 logins, representing fewer than one in ten users. The remaining CHWs were distributed at mid- to low frequency, with the most common group logging in 5–14 times.

To explore this variation further, we fitted an exploratory mixed-effects model. The model was not pre-specified and should be interpreted as a descriptive analysis of usage patterns rather than a full explanatory model of app use. The model results showed that usage patterns varied not only by district and timing of implementation but were also strongly clustered within teams, suggesting that unmeasured team-level factors impacted engagement. Full details are provided in the appendices (S3 File).

### Data Completeness and Triaging of Participants

AitaHealth enabled CHWs to systematically record household information, supporting an assessment of data completeness and the generation of outcomes. Table 6 Reports include two indicators: (i) completed household assessments, the number and proportion of households where the relevant field was filled in the tool regardless of response, and (ii) triaged participants, the number and proportion of households where at least one member met the specified condition.

**Table 6:** Data completeness and triaged participants.

**Household characteristics (n=2452)**				
*Household triaged*	1945	*79.3%*		
*Has GPS coordinates*	1866	*76.1%*		
**Tuberculosis indicators (n=2129)**				
*≥1 member currently on TB treatment*	2049	*96.2%*	211	*8.6%*
*≥1 member completed TB treatment (past 12 months)*	2045	*96.1%*	123	*5.0%*
*≥1 member diagnosed, not on treatment (past 6 months)*	2043	*96.0%*	68	*2.8%*
*≥1 member defaulted after TB diagnosis*	2043	*96.0%*	92	*3.7%*
*≥1 member had TB symptoms*	2016	*94.7%*	152	*6.2%*
**HIV, Home-based care & Maternal Health (n=2129)**				
*≥1 member required home-based care*	1998	*93.9%*	76	*3.1%*
*≥1 member HIV-exposed*	1998	*93.9%*	-	-
*HIV test requested for more than one member*	2027	*95.2%*	238	*9.6%*
*≥1 pregnant member (ANC)*	2012	*94.5%*	142	*5.8%*
*≥1 member receiving post-natal care*	2001	*94.0%*	78	*3.2%*

^*1*^ *Completed household assessments = number (and %) of households where the relevant field was filled in the tool, regardless of the response entered.*

^*2*^ *number (and %) of households where at least one member actually met the specified indicator (e.g., on TB treatment, reported TB symptoms)*

Data completeness was consistently high when AitaHealth was used. In over 94% of the 2,452 households, CHWs completed TB-related fields for at least one household member, including treatment status, symptom screening, and treatment history. Maternal and child health indicators were also well captured in more than 93% of households. HIV-related fields, including test requests and exposure status, were similarly complete. Household-level fields such as triage and GPS coordinates were slightly less complete, documented in 79.3% and 76.1% of households, respectively.

From these completed assessments, CHWs were able to triage a range of outcomes, reflecting AitaHealth’s ability to support multi-disease data collection within a single outreach visit. For example, in 8.6% of households, at least one member was currently on TB treatment, while smaller proportions included members who had completed treatment (5.0%), defaulted (3.7%), or reported TB symptoms (6.2%). Non-TB outcomes were also identified: 5.8% of households included a pregnant woman, 3.2% reported postnatal care, and 9.6% had more than one member requesting an HIV test. These findings demonstrate that AitaHealth achieved high data completeness while also enabling CHWs to generate actionable triage outcomes across multiple health areas during a single household visit.

### Overall summary of qualitative and quantitative results

The qualitative findings from the FGDs showed that CHWs valued AitaHealth for improving efficiency, data security, and the credibility of household data recording. The IDIs provided a broader perspective on implementation, with team leaders and key stakeholders describing AitaHealth as useful when the platform was functional, available, and aligned with routine fieldwork. The quantitative findings reflected this pattern of use, with CHWs using AitaHealth to successfully record data for 2,452 households and 10,649 household members across the two districts, and to timestamp 83.6% of individual entries.

Although the overall usage figures indicated substantial household-level data capture, the qualitative findings showed that CHWs consistently faced practical difficulties with AitaHealth during routine fieldwork. In the FGDs, CHWs described unreliable devices, weak technical support, connectivity problems, and safety concerns as barriers to routine use. The IDIs similarly highlighted constraints linked to support systems, supervision, and working conditions. These findings aligned with the quantitative pattern of uneven engagement: 48.5% of CHWs logged in fewer than 15 times during implementation, while only 9.7% logged in more than 75 times. AitaHealth was also used on fewer than half of active implementation days in both districts.

The qualitative findings further showed that CHWs viewed AitaHealth as helpful for structuring household visits and documenting TB contact-tracing information when they could use the platform. This was reported mainly in the FGDs, while the IDIs provided context on how AitaHealth fitted into routine community-based work. The quantitative findings were consistent with this pattern, showing that TB-related fields were completed in more than 94% of households when AitaHealth was used, including treatment status, symptom screening, and treatment history.

Overall, qualitative results from FGDs and IDIs showed CHWs and stakeholders valued AitaHealth but faced barriers to consistent use. Quantitative data indicated substantial household-level capture, uneven engagement, and high TB field completeness when used. AitaHealth aided TB contact tracing documentation, though its routine use varied by setting and conditions.

## Discussion

AitaHealth was well-received by CHWs when operational and was perceived to improve efficiency, data accuracy, and referral processes. Supervisors and stakeholders valued its real-time monitoring capabilities and automated reporting functions. Feasibility, however, was constrained by unreliable hardware, limited technical support, and insecure working conditions, which directly affected implementation fidelity. Despite the relative acceptability evident in qualitative interviews, the tool was used on fewer than half of the active days. We interpret this dissonance as a pragmatic, task-level choice where CHWs used the tool when it added clear value and reverted to paper when it created challenges that disrupted their work. This illustrates that even tools views favourably will not be used consistently unless operational conditions enable ease of use.

mHealth technologies are now established as functional support mechanisms for community-based care activities, particularly in TB contact tracing [20,35,36]. Evidence of their effectiveness in routine settings and an understanding of the enabling conditions remain limited. Our evaluation addresses this by assessing AitaHealth’s acceptability, feasibility, and fidelity in CHW workflows. Such broader implementation-focused perspectives are often missing from most evaluations. A recent systematic review noted that over half of implementation studies only evaluated acceptability, with just over a third assessing feasibility and fidelity and fewer than a quarter examining more core outcomes [37]. This narrow perspective hinders the understanding of real-world interventions. By analysing these outcomes together, our study clarifies not only whether AitaHealth was used but also why its use, thus providing insight into the conditions necessary for the effective implementation of digital tools.

CHWs consistently found AitaHealth useful because it supported their tasks, affirming that mHealth tools are more likely to be accepted when they support routine responsibilities [38–40]. However, ease of use was limited, not by the tool itself, but by CHWs’ digital literacy and confidence in using it. The 2023 CHW and Outreach Team Leader Skills Audit revealed that nearly half of CHWs still lack a high school qualification, and many struggled to engage meaningfully with training [41]. These constraints reveal a broader structural problem which exacerbates in systems like mHealth. Introducing and using mHealth technologies will thus remain challenging if these circumstances persist, leading to inconsistent adoption. Targeted or differentiated training may offer short-term improvements for staff members who are more challenged, but it is resource-intensive and difficult to sustain over time. We support the Skills Audit recommendations for minimum education requirements while also increasing the capacity of CHWs already in the system [41], a strategy that is both equitable and non-exclusionary. While these reforms are not specific to mHealth, they reflect a broader shift toward digital readiness that will continue to affect adoption. Future programmes will need to address this, either through recruitment or sustained, practical training.

Feasibility challenges in our study reflect patterns observed elsewhere, where digital tools are often well-received by CHWs but remain difficult to use consistently in routine conditions [42]. Although South Africa’s CHW programme and infrastructure differ from those in Uganda, Tanzania, and Malawi, similar challenges, including poor connectivity, device failures, and stigma, despite user acceptability, reinforce the broader relevance of our findings [10,40,43]. This disconnect is well established in implementation science, where acceptability alone does not guarantee adoption [22,37], a point clearly articulated by Dimanopoulos and colleagues, who note that “acceptability may predict adoption, [but] it does not guarantee engagement”, particularly when contextual and structural barriers persist [44]. Broader implementation frameworks also caution that adoption unfolds gradually, requiring time, policy alignment, and sustained infrastructural support to become embedded in daily workflows [45,46]. It is therefore plausible that our evaluation, like others, captured an early phase of implementation before adoption had stabilised. Nonetheless, these challenges are no longer unforeseen; future mHealth programmes must anticipate them and proactively plan to mitigate their effects.

Device quality was a recurring challenge, with CHWs often experiencing battery failures, overheating, and fragile hardware, which undermined consistent use. In our study, devices degraded rapidly, likely reflecting their overall quality and the demands of daily fieldwork. For many CHWs, this was their first time using such a device. Although we did not track non-study usage, it is possible they used them for personal purposes, which would have added to the wear and tear. These experiences emphasise the need for structured device management, including regular inventories, replacement plans, and accountability systems. However, approaches that entirely restrict personal use may be counterproductive, as CHWs valued these devices and incorporated them into their daily routines. A more effective approach, we believe, is to provide durable equipment and establish systems to manage and monitor them responsibly, while maintaining CHWs’ motivation to use digital tools in their work.

The personal implications of outreach work are crucial to community-based interventions. South Africa’s high crime rates disproportionately affect women, who constitute most CHWs, increasing the risks tied to carrying valuable devices like tablets. Our study revealed persistent security concerns among CHWs, yet no easy solutions to secure their working environments were evident. Although no incidents of theft or harm were formally reported to the study team, we acknowledge that we could have been more proactive in tracking such events. This was a missed opportunity and represents a key limitation in capturing risks associated with outreach fieldwork. Interventions requiring physical mobility must go beyond general safety guidance to include structured systems for logging safety incidents and institutional mechanisms for risk mitigation. These concerns, though contextual, are integral to outreach work and should be planned systematically.

Our study emphasised the longstanding labour challenges faced by CHWs. As Malatji, Griffiths [47] noted, CHWs strive for role legitimacy and fair pay. In our study, these concerns were reflected in participants’ accounts of remuneration and recognition, including one CHW who noted that “even in my family they think that I have a better salary since I am now using a tablet.” In this broader context, CHWs also expressed frustration, feeling that their efforts primarily benefited researchers without yielding tangible returns. Some described the tablet as the only visible source of recognition for their work; an observation echoed in findings from Malawi, where digital tools similarly became proxies for professional identity [10]. This reflects a troubling pattern where CHWs remain consistently undervalued across different settings despite their vital role in delivering public health services. More worryingly, some saw remote supervision as affirming, not because it strengthened support or accountability, but because it helped counter the persistent scepticism surrounding their commitment. While efforts by the government and unions to professionalise the cadre have begun, progress remains uneven. To realise the full value of mHealth interventions, policymakers must go beyond improving digital platforms. They must also improve conditions for people who use them by providing formal employment, fair compensation, professional recognition and the structural integration of CHWs into the public health workforce.

Fidelity in this study reflected partial implementation rather than rejection. CHWs incorporated AitaHealth into their outreach but used it inconsistently, not because of poor design or user resistance but because the conditions needed for sustained engagement were not in place. Challenges such as unreliable devices, limited support systems, and structural constraints created an environment that made sustained regular use difficult. Where AitaHealth was used, it improved workflow structure and supported high data completeness, demonstrating its functional potential; however, the broader system did not consistently act on the information produced. This highlights an important implementation insight: fidelity must be interpreted not only in terms of adherence to use but also as a reflection of system responsiveness. Addressing these gaps will require more than training or technical adjustments. It calls for implementation strategies that integrate digital tools into service delivery systems to enable their full functionality.

Implementation experiences varied between Ekurhuleni (urban) and uMkhanyakude (rural), demonstrating how local context and team dynamics shape digital engagement. CHWs in uMkhanyakude used AitaHealth more often, despite facing weaker infrastructure and more challenging working conditions. This was unexpected, as higher usage was expected in the urban setting. Our mixed-effects model suggested that these differences may have been influenced by unmeasured team-level factors, such as supervision quality or informal leadership, rather than solely by individual or household characteristics. However, this increased use did not determine sustainability. AitaHealth did not continue in either district; it was discontinued in uMkhanyakude due to lack of funding, and in Ekurhuleni, it was replaced with a free transition to CommCare, highlighting the absence of long-term financial planning as a central barrier to sustainability. To attract future investment, programmes need to demonstrate not only uptake but also generate evidence aligning with funder priorities. This includes not only usage data but also economic evidence to inform decisions on resource use and allocation.

Supervisors shared many concerns raised by CHWs but viewed them from a broader programme perspective, whereas CHWs mainly judged the tool on its performance in the field. Although we cannot assess the extent to which their views influenced the continued implementation of mHealth in Ekurhuleni, securing supervisor buy-in is crucial for adopting and scaling these initiatives. Research indicates that middle managers play an important role in translating adoption decisions into practice by coordinating resources, motivating staff, and monitoring progress [48]. Recent South African studies highlight gaps in leadership readiness that could hinder implementation [49]. In our study, Ekurhuleni continued implementing mHealth tools as long as funding remained available, but the project in uMkhanyakude ended once funding ran out. These patterns suggest that funding enables continuation, but sustainable use relies on managerial support.

Our study faced limitations due to a reduced scale of implementation. We worked with CHWs embedded in government services to reflect real-life conditions and to improve sustainability. While this strengthened external validity, it also required time for training and coordination, which delayed our expansion within the timeline. A more extended preparatory phase might have helped.

We also struggled to link household contact-tracing data to clinic-level outcomes, such as TB diagnoses. While facility statistics were available, attributing outcomes to AitaHealth use was challenging because follow-up actions often rely on other personal and clinic factors beyond the control of CHWs. This reflects a broader issue with many interventions, including some mHealth, which can enhance data quality and efficiency; however, their impact on clinical outcomes may be indirect unless systematic support is provided for the entire cascade [50]. Measuring programmatic impact would have required a larger design implemented over a more extended period, such as ZAMSTAR, a cluster-randomised trial in Zambia and South Africa that evaluated community-wide household interventions with population-level outcomes [51]. In contrast, our study was designed to generate foundational evidence on feasibility, acceptability, and fidelity under routine conditions, as a necessary precursor to larger-scale trials or programs capable of assessing epidemiological impact. More integrated approaches connecting CHWs, clients, and facilities may be needed to bridge this gap. This scale and length limitation also meant we could not formally validate data accuracy by comparing digital entries with paper-based clinic records, due to resource and access constraints.

In Ekurhuleni, qualitative data collection continued after CHWs transitioned from AitaHealth to the CommCare platform, which retained many of AitaHealth’s original workflows but was developed and managed by a different provider. Although participants were asked to reflect specifically on AitaHealth, their responses may have been influenced by subsequent use of CommCare. This limitation is worth acknowledging; however, the transition itself may be seen as a positive indicator of adoption and sustainability, as it demonstrates that the core principles and workflows introduced by AitaHealth were preserved and carried forward to the new platform.

Lastly, our mixed-effects model was exploratory and not prespecified. While it provided useful descriptive insight into patterns of AitaHealth use, we acknowledge that the model would have been strengthened by including additional CHW-level variables, such as age, years of service, prior training, and digital experience. These variables could not be included because they were collected only from CHWs who participated in the FGDs and were therefore unavailable for all CHWs who used AitaHealth. Future studies should collect these variables for all CHWs to support a fuller assessment of the factors influencing digital tool use.

## Conclusion

This evaluation demonstrates that mHealth interventions, such as AitaHealth, have potential and are may be acceptable to users. In our study, AitaHealth improved workflow structure and supported high data completeness, suggesting that digital tools have tcan strengthen outreach services and lay the groundwork for improved outcomes. However, acceptability and functionality alone are not enough. Implementation success depends on the systemic contexts under which these tools are deployed. For policymakers, this means that realising the full value of mHealth will require investing in the broader systems, technical, operational, and workforce-related, that enable consistent and effective use.

## Supporting information

S1 File Focus Group Discussion guides. (PDF)

S2 File In-depth interview data collection guides. (PDF)

S3 File Exploratory mixed-effects model. (PDF)
