# Supplementary material for: Evaluating the acceptability, feasibility, and implementation fidelity of a multipurpose mobile health (mHealth) app used for Tuberculosis (TB) contact tracing by Ward-Based Outreach Teams (WBOTs) in South Africa
Source: PLOS Glob Public Health. 2026 Aug 31;6(8):e0007120. doi: 10.1371/journal.pgph.0007120 (PMC13528960; doi:10.1371/journal.pgph.0007120)
Supplement: Supplementary file 1 [file pgph.0007120.s001.pdf]

STUDY NUMBER:□□□-□-□-□□□□-□-□□□AUR5-7-182B

FOCUS GROUP DISCUSSION GUIDE: COMMUNITY CARE GIVERS

Phase II: Optimizing the efficiency of household contact tracing for TB control in South Africa

Introduction and Ground Rules

1. Obtain written informed consent first from all participants, before any data are collected.

2. Interviewer to introduce self. Thank you for taking the time to meet with us today. Our names are [*insert names*] ______________________________ and we would like to talk to you about TB contact tracing. We are doing this project to understand how we may improve TB contact tracing in the community, and particularly to see how feasible the intervention was to deliver TB contact tracing activities. We are interested in your views regarding your experience, thoughts and suggestions with the TB contact tracing intervention. We want you to be as open and honest when answering. There are no right or wrong answers in this discussion. Please feel free to tell us what you think, regardless of whether you agree or disagree with what you hear. We don’t expect everyone to share the same opinions. It is very important that we hear all your opinions.

3. Interviewer to explain the ground rules and terms of confidentiality for the focus group discussion:

    a. The participant does not have to answer any question they do not want to.
    b. The information you share will be handled in confidence (in secret).
    c. When we report back on the information collected in this discussion, your comments will not be able to be linked to you specifically.
    d. We ask that you also agree not to share anything discussed in this room with others. Although we request that you maintain confidentiality, there is no way we can guarantee that this request will be respected.

4. The discussion should take between one and two hours. We will have a break somewhere in the middle.

5. Interviewer to inform the participants that the focus group discussion will be tape recorded to make sure that all themes are captured. Turn the tape on and ask for verbal permission again to tape record, while the tape is running to verbally capture consent (this is a double check against the written consent). We will be recording the session because we don’t want to miss any of your comments. Although one of us may take some notes while we talk, we

can't write fast enough to get everything down on paper. As we are recording, please try to speak loudly so that we don't miss your comments.

Themes to be explored

(i) Knowledge of the TB contact tracing intervention model
(ii) Feasibility of implementing the intervention (barriers and motivators)
(iii) Factors that influence the sustainability of the intervention

<u>Time started (HHMM)</u>:

Questions

1. **Knowledge of the TB contact tracing intervention model**
   - *Can you describe to us your understanding of the TB contact tracing intervention? Please highlight to us the components, activities and cadre of staff involved with carrying out the intervention.*
   - *Can you give us a brief description of your role with the TB contact tracing intervention activities?*
   - *Please describe the training that you received on the TB contact tracing intervention activities?*
   - *In your opinion, please describe how the TB contact tracing intervention influenced your daily role?*

2. **Feasibility of implementing the intervention (barriers and motivators)**

   - *How did you feel about carrying out these activities for the TB contact tracing intervention?*
   - *Can you tell us about the resources needed and what was available for implementation of the TB contact tracing intervention activities?*
   - *Please describe the reporting and communication strategies used during implementation of the TB contact tracing intervention.*
   - *In your opinion how easy was it for you to implement the TB contact tracing intervention activities?*
   - *In your opinion how difficult was it for you to carry out the TB contact tracing intervention activities?*

3. **Factors that influence the sustainability of the intervention**
   - *Explain any general, positive or negative experiences of carrying out the TB contact tracing intervention activities?*
   - *Please describe the community's reaction to the TB contact tracing intervention. What do you think are the thoughts and feelings from community members toward the TB contact tracing intervention activities? (Probe on support or resistance from community)*
   - *Please describe the reaction to the TB contact tracing intervention at the facility level. What do you think are the thoughts and feelings from clinical*

staff based at the facilities toward the TB contact tracing intervention activities? (Probe on support or resistance from the facility)

- *How do you feel about CCGs being involved in the TB contact tracing intervention activities? What would be reasons for and against this?*
- *Can you describe to us the motivators or challenges that community members could experience as part of the TB contact tracing intervention?*

Any other comments

Are there any final thoughts you have about TB household contact tracing?

End of session

Now we have come to the end of our discussion. Thank you for your active participation. If you have any questions about your study participation, please contact us. Thank you.

Time ended (HHMM):
