# Supplementary material for: Evaluating the acceptability, feasibility, and implementation fidelity of a multipurpose mobile health (mHealth) app used for Tuberculosis (TB) contact tracing by Ward-Based Outreach Teams (WBOTs) in South Africa
Source: PLOS Glob Public Health. 2026 Aug 31;6(8):e0007120. doi: 10.1371/journal.pgph.0007120 (PMC13528960; doi:10.1371/journal.pgph.0007120)
Supplement: Supplementary file 2 [file pgph.0007120.s002.pdf]

STUDY NUMBER:□□□-□-□-□□□□-□-□□□AUR5-7-182B

INTERVIEW GUIDE:

DEPARTMENT OF HEALTH KEY STAKEHOLDERS/ FACILITY-BASED STAFF

Phase II: OPTIMIZING THE EFFICIENCY OF HOUSEHOLD CONTACT TRACING FOR TB CONTROL IN SOUTH AFRICA

Introduction

Staff to say out loud before starting interview

- Thank you for agreeing to take part in our research
- We are interested in knowing about TB contact tracing. There are no right or wrong answers.
- <u>You do not have to answer any questions if you don't want to.</u> Just say pass and I will move to the next question.
- Your answers to these questions may help us improve TB services.
- All of your answers will be kept confidential. Confidential means we will not tell anyone your answers outside the research group.

Introduction and Ground Rules

1. Obtain written informed consent first, before any data are collected.

2. Interviewer to introduce self. Thank you for taking the time to meet with us today. Our names are [*insert names*] __________ and we would like to talk to you about the TB contact tracing intervention. We are doing this project to understand your role, thoughts, experiences and perceptions of the TB contact tracing intervention and particularly to see whether this intervention could be sustained within the TB programme. We want you to be as open and honest when answering. There are no right or wrong answers in this discussion. Please feel free to tell us what you think.

    - Interviewer to explain the ground rules and terms of confidentiality for the interview.
    - The participant does not have to answer any question they do not want to.
    - The information you share will be handled in confidence. (in secret)
    - When we report back on the information collected in this discussion, your comments will not be able to be linked to you specifically.
    - We ask that you also agree not to share anything discussed in this room with others, however we cannot guarantee that this request will be respected

3. The interview should take about 2 hours

4. Interviewer to inform the interviewee that the in-depth interview will be tape recorded to make sure that all themes are captured. Turn the tape on and ask for verbal permission again to tape record, while the tape is running to verbally capture consent (this is a double check against the written consent). We will be recording the session because we don't want to miss any of your comments. Although one of us may take some notes while we talk, we can't write fast enough to get everything down on paper. As we are recording, please try to speak loudly so that we don't miss your comments.

Themes to be explored

I. Feasibility of implementing the intervention (motivators and barriers)
II. System level challenges of delivering intervention
III. Factors that influence the sustainability of the intervention

Time started (HHMM):

Questions

1. **General**

- What is the title of your current position?
- How long have you held this position?
- How are TB services delivered at your level with regard to the intervention? (i.e. District, Provincial, National)

2. **Feasibility of implementing the intervention (motivators and barriers)**

- *Can you tell us who the cadre of staff are that are delivering TB contact tracing intervention services?*
- *Can you describe the reporting lines/ supervision among the cadre of staff delivering TB contact tracing intervention services?*
- *What is your opinion of the cadre of staff who are conducting these activities? Probe on suitability and competency*
- *Can you describe the training and resources that staff received to perform the TB contact tracing intervention? In your opinion, are there sufficient resources and adequate training available to perform the TB contact tracing intervention? How do you think availability of resources and training could be changed to be more effective? Explain.*
- *Which individuals or households are targeted for the intervention? Are certain patient groups prioritised?*
- *Do you believe that the TB contact tracing intervention is being delivered optimally? If yes or no, explain.*
- *Can you describe the benefits of the TB contact tracing intervention and should we continue with this?*
- *How can tracing of TB in the community be sustained using this intervention?*
- *Can you describe the challenges of the TB contact tracing intervention and should we continue with this?*
- *How can tracing of TB in the community be improved using this intervention?*

3. **System level challenges of delivering intervention**

- *Can you describe what was previously done as standard of care for household contact tracing?*
- *Can you describe how the intervention is different to the standard of care?*
- *Please describe the changes that emerged when the intervention was implemented.*
- *Please elaborate on the positive changes of the intervention. How do you think the positive changes could be sustained?*

- *Please elaborate on the negative changes of the intervention. How do you think the negative changes could be addressed?*
- *Can you describe to us what systems are in place that monitors the TB contact tracing intervention activities? In your opinion what are the challenges and positive factors of these systems (M&E, etc.) in monitoring and/or improving TB contact tracing intervention activities? Explain.*

4. **Factors that influence the sustainability of the intervention**

- *Please describe to us the expectations that you may have had about the TB contact tracing intervention model?*
- *How different is the current intervention model compared to your initial expectations and how do you think this model could be more effective?*
- *Can you describe any gaps which exist in the way the intervention is being delivered currently?*
- *Can you describe the positive and negative aspects of the cadre of staff responsible for delivery of the intervention?*
- *Can you describe the relationship between the facility and cadre of staff delivering the intervention?*

Any other comments

Are there any final thoughts you have about TB household contact tracing intervention?

End of session

Now we have come to the end of our discussion. Thank you for your participation. If you have any questions about your study participation, please contact us. Thank you.

Time ended (HHMM):
