# Supplementary material for: Evaluating the acceptability, feasibility, and implementation fidelity of a multipurpose mobile health (mHealth) app used for Tuberculosis (TB) contact tracing by Ward-Based Outreach Teams (WBOTs) in South Africa
Source: PLOS Glob Public Health. 2026 Aug 31;6(8):e0007120. doi: 10.1371/journal.pgph.0007120 (PMC13528960; doi:10.1371/journal.pgph.0007120)
Supplement: Supplementary file 3 [file pgph.0007120.s003.pdf]

Supplementary File 3: Exploratory mixed-effects model

During our analysis, we observed considerable variation in CHW use of AitaHealth, with some logging in frequently and nearly half logging in fewer than 15 times over 9–12 months. To better understand whether this variation was due to structural, individual, or contextual factors, we conducted an exploratory mixed-effects regression model that was not prespecified. We examined whether the variation clustered at the team level, indicating unmeasured influences such as supervision, peer support, and local management. The model helped us infer organisational dynamics that were not directly collected. Our focus was on CHWs' outreach days, with fixed effects for district (Ekurhuleni as the reference), months since launch, usage span, and household visits (per 10) to capture context, timing, exposure, and workload. A mixed-effects model with team as a random effect accounted for clustering, as CHWS were organised into teams.

**Predictor**	**Estimate (β)**	**Std. Error**	**95% CI**	**p-value**
District (ref: Ekurhuleni)	0.84	0.29	0.27 – 1.41	0.004
Start date (months since launch)	-0.05	0.02	-0.09 – -0.01	0.018
Usage span (months)	0.12	0.03	0.06 – 0.18	<0.001
Household visits (per 10)	0.07	0.01	0.05 – 0.09	<0.001
Random effect (team)			Variance = 0.21	

The model demonstrates that variation in usage was systematically linked to both individual- and team-level factors. The positive coefficient for the rural district (β = 0.84)

shows that CHWs in uMkhanyakude recorded notably more outreach days than those in Ekurhuleni, indicating that context influences uptake, not just enthusiasm. Later start dates (β = –0.05/month) were associated with decreased usage as devices aged or support diminished. In comparison, longer exposure (β = 0.12/month) and higher workload (β = 0.07/10 visits) were associated with more recorded days, emphasising the importance of opportunity and consistency. A random-effect variance of 0.21 indicates that usage was clustered within teams in patterns not explained by district, timing, exposure, or workload, suggesting that team practices, peer dynamics, and organisational factors impact AitaHealth use.

Our model was exploratory, so its findings should be approached with caution. Nevertheless, it indicates that individual factors did not fully account for the variation in usage, with some differences clustering at the team level. Although the model cannot identify the precise causes, it highlights the importance of unmeasured dynamics beyond the individual. Future studies should therefore collect variables such as supervision, peer support, and team organisation, as these will be crucial to understanding and enhancing consistent digital tool use.
