# Supplementary material for: Evaluating the acceptability, feasibility, and implementation fidelity of a multipurpose mobile health (mHealth) app used for Tuberculosis (TB) contact tracing by Ward-Based Outreach Teams (WBOTs) in South Africa
Source: PLOS Glob Public Health. 2026 Aug 31;6(8):e0007120. doi: 10.1371/journal.pgph.0007120 (PMC13528960; doi:10.1371/journal.pgph.0007120)
Supplement: Supplementary file 5 [file pgph.0007120.s005.docx]

**15 May 2026**

Editor-in-Chief

1265 Battery Street, Suite 200

San Francisco, CA 94111

United States

Dear Sir/Madam,

We are pleased to resubmit our revised manuscript titled “Evaluating the acceptability, feasibility, and implementation fidelity of a multipurpose mobile health (mHealth) app used for Tuberculosis (TB) contact tracing by Ward-Based Outreach Teams (WBOTs) in South Africa.” We sincerely thank the reviewers for their constructive and insightful feedback. Their comments have helped us improve the manuscript, strengthen our arguments, and clarify the overall presentation. We have carefully addressed each point and believe these revisions significantly enhance the paper. As requested, we have included both a tracked-changes version and a clean version of the revised manuscript.

We also took the opportunity to proofread the manuscript and improve some sections not raised by the reviewer, which we believed would make it clearer. All the changes are reflected in the tracked changes.

**Reviewer #4:** This study evaluated an mHealth application (AitaHealth) for household contact tracing and described the acceptability, feasibility, and implementation fidelity of the mHealth approach. The authors have responded constructively to the first round of reviews. I have several comments and questions for the authors’ consideration.

1. **The manuscript would benefit from a clearer description of how AitaHealth was specifically used for TB contact tracing. The tool reads primarily as a general data collection platform.**

*Thank you for this comment. We have revised the manuscript to clarify how AitaHealth was used for TB contact tracing. AitaHealth was a general-purpose data-collection platform with no disease-specific focus, but it allowed users to customise modules according to programme needs. In this study, we worked with the developers to strengthen the TB contact-tracing questions and workflows to support more consistent completion by Department of Health staff. Because these staff also delivered broader household-based primary care services, we retained the other routine modules rather than creating a contact-tracing-only tool. The revised manuscript now clarifies that the study focused on optimising the TB contact-tracing component within a broader household data-collection platform.*

***The revised manuscript text now reads as follows:***

*AitaHealth was a general-purpose data-collection platform with no disease-specific focus. It allowed users to customise modules to programme needs. For our study, we worked with the developers to strengthen the TB contact-tracing questions and workflows, enabling Department of Health staff to complete household contact tracing more consistently. We retained other routine household-care modules, such as maternal health, because the same staff also delivered broader primary care services during household visits. Thus, the intervention focused on optimising the TB contact-tracing component within a broader household data-collection platform.*

2. **CHWs trained / training completion rate: Listed as a fidelity indicator with "% trained, completion rates" as the intended metric. This is not reported in the quantitative results. Only a narrative description of training is provided.**

*Thank you for this comment. We agree that the CHW training completion rate should be reported in the quantitative results, as it was listed as a fidelity indicator in the evaluation framework. We have now added this metric to the relevant paragraph, but not in the table, as we still feel it is sufficient even if stated in the opening paragraph. CHWs were expected to complete the training as part of their work responsibilities before using AitaHealth; it was not optional. However, we recognise that this indicator remains important for assessing fidelity to the planned implementation process. Required training can still demonstrate high implementation fidelity when delivered to and completed by all intended users. All CHWs expected to use AitaHealth completed the required training before implementation, resulting in a 100% training completion rate.*

***We also added this sentence in the fidelity section.***

*All CHWs completed their training before using AitaHealth, achieving a 100% completion rate because it was integrated into their routine tasks and made mandatory.*

3. **The results remain largely siloed: qualitative findings are presented in one section and quantitative findings in another. Given that this is a mixed-methods study, an integrated results section would strengthen the paper.**

*Thank you for this comment. We agree that the mixed-methods design is strengthened by presenting the qualitative and quantitative findings more cohesively. We have added a Results section that demonstrates how the qualitative and quantitative findings align. This section links CHW and stakeholder accounts from the FGDs and IDIs with the quantitative findings on household-level data capture, routine engagement, and completeness of TB-related fields.*

***The following revision is now included as the last paragraph in the results section***

*The qualitative findings from the FGDs showed that CHWs valued AitaHealth for improving efficiency, data security, and the credibility of household data recording. The IDIs provided a broader perspective on implementation, with team leaders and key stakeholders describing AitaHealth as useful when the platform was functional, available, and aligned with routine fieldwork. The quantitative findings reflected this pattern of use, with CHWs using AitaHealth to successfully record data for 2,452 households and 10,649 household members across the two districts, and to timestamp 83.6% of individual entries.*

*Although the overall usage figures indicated substantial household-level data capture, the qualitative findings showed that CHWs consistently faced practical difficulties with AitaHealth during routine fieldwork. In the FGDs, CHWs described unreliable devices, weak technical support, connectivity problems, and safety concerns as barriers to routine use. The IDIs similarly highlighted constraints linked to support systems, supervision, and working conditions. These findings aligned with the quantitative pattern of uneven engagement: 48.5% of CHWs logged in fewer than 15 times during implementation, while only 9.7% logged in more than 75 times. AitaHealth was also used on fewer than half of active implementation days in both districts.*

*The qualitative findings further showed that CHWs viewed AitaHealth as helpful for structuring household visits and documenting TB contact-tracing information when they could use the platform. This was reported mainly in the FGDs, while the IDIs provided context on how AitaHealth fitted into routine community-based work. The quantitative findings were consistent with this pattern, showing that TB-related fields were completed in more than 94% of households when AitaHealth was used, including treatment status, symptom screening, and treatment history.*

*Overall, qualitative results from FGDs and IDIs showed CHWs and stakeholders valued AitaHealth but faced barriers to consistent use. Quantitative data indicated substantial household-level capture, uneven engagement, and high TB field completeness when used. AitaHealth aided TB contact tracing documentation, though its routine use varied by setting and conditions.*

4. **Did the authors consider including other drivers of app use, such as CHW age, years of service, and training, in the mixed effects model? Could the authors clarify whether these variables were considered for inclusion in the model, and if so, why they were excluded? If the data were not available, this should be acknowledged as a limitation.**

*Thank you for this comment. Indeed, CHW age, years of service, prior training, and related individual characteristics could help explain variation in app use. These factors were considered conceptually but were not available for all CHWs. Detailed demographic and work-experience data were collected only from CHWs who participated in the FGDs, not from the full group of CHWs who used the app. We therefore did not include these variables in the mixed-effects model, as doing so would have restricted the analysis to the FGD subgroup and would not have accurately reflected the full implementation cohort. We acknowledge this as a limitation and have included this statement in the discussion for future researchers to consider.*

***The revised manuscript text now reads as follows in the results:***

*To explore this variation further, we fitted an exploratory mixed-effects model. The model was not pre-specified and should be interpreted as a descriptive analysis of usage patterns rather than a full explanatory model of app use. The results showed that usage patterns varied not only by district and implementation timing but were also strongly clustered within teams, suggesting that unmeasured team-level factors influenced engagement.*

***And in a section of the discussion, as follows:***

*Lastly, our mixed-effects model was exploratory and not prespecified. While it provided useful descriptive insight into patterns of AitaHealth use, we acknowledge that the model would have been strengthened by including additional CHW-level variables, such as age, years of service, prior training, and digital experience. These variables could not be included because they were collected only from CHWs who participated in the FGDs and were therefore unavailable for all CHWs who used AitaHealth. Future studies should collect these variables for all CHWs to support a fuller assessment of the factors influencing digital tool use.*

5. **CHWs used the app on approximately 12% of weekends due to their work commitments, which was acknowledged in the response to reviewers but not clear in the revised manuscript.**

*Thank you for this comment. Weekend use of AitaHealth was included in the revised manuscript submitted with our previous response, but we recognise that our response letter did not clearly indicate where this information appears. The weekend-use findings are reported in the Results subsection “AitaHealth Use on Active Days” and in Table 4. In this subsection, the manuscript states that AitaHealth was used on just over half of weekdays in both sites: 54.4% in Ekurhuleni and 50.2% in uMkhanyakude. The same subsection also reports weekend use: CHWs used the tool on 12.1% of weekends in Ekurhuleni and 11.4% in uMkhanyakude. Table 4 provides the related breakdown of active days, outreach days, weekend use, and implementation duration.*

6. Each FGD is described as including 12 participants, yet the totals (25 in Ekurhuleni, 43 in uMkhanyakude across two FGDs each) do not consistently add up to 12 per group. This should be clarified.

We corrected the manuscript and Table 2 for consistency, clarifying that each FGD comprised 12 participants. Four FGDs were conducted, yielding 24 participants from Ekurhuleni and 24 from uMkhanyakude. Additionally, 10 participants completed IDIs, bringing the total qualitative sample to 58 (48 FGD and 10 IDI). Both the manuscript and Table 2 now reflect these figures.

7. Where possible, the authors may wish to consider including key quantitative findings in the abstract

Thank you. The abstract has been revised.

Yours sincerely,

Don Mudzengi

Email:	[dmudzengi@auruminstitute.org](mailto:dmudzengi@auruminstitute.org)

Cellphone:	+27 (0) 83 369 7946

Landline:	+27 (0) 10 590 1388
