# Supplementary material for: Evaluating the acceptability, feasibility, and implementation fidelity of a multipurpose mobile health (mHealth) app used for Tuberculosis (TB) contact tracing by Ward-Based Outreach Teams (WBOTs) in South Africa
Source: PLOS Glob Public Health. 2026 Aug 31;6(8):e0007120. doi: 10.1371/journal.pgph.0007120 (PMC13528960; doi:10.1371/journal.pgph.0007120)
Supplement: Supplementary file 6 [file pgph.0007120.s004.docx]

**26 September 2025**

Editor-in-Chief

1265 Battery Street, Suite 200

San Francisco, CA 94111

United States

Dear Sir/Madam,

We are pleased to submit our revised manuscript titled *“Evaluating the acceptability, feasibility, and implementation fidelity of a multipurpose mobile health (mHealth) app used for Tuberculosis (TB) contact tracing by Ward-Based Outreach Teams (WBOTs) in South Africa.”* We greatly appreciate the constructive and insightful feedback provided by the reviewers. Their comments have helped us refine the manuscript, strengthen our arguments, and improve its overall clarity. We have carefully addressed each point raised and believe the revisions substantially enhance the paper. As requested, we have attached both a tracked-changes version and a clean version of the revised manuscript.

**Reviewer #1:** Overall, good compliance to PLOS publication criteria. Just to minor comments for authors to consider, maybe good to mention what tools (if any) were used for both qualitative and quantitative data management, and were there any ethical considerations for the quantitative data used? Please clarify.

*We thank the reviewer for this helpful suggestion. For quantitative analysis, we used Stata version 16 and Microsoft Excel. For qualitative data, we combined Quirkos with manual coding: Quirkos was used to organise codes, while manual coding allowed cross-checking and consensus during thematic analysis. The study was approved by the University of the Witwatersrand Human Research Ethics Committee and the LSHTM Ethics Committee, with all quantitative data de-identified and stored securely.*

*The Quirkos responses are now provided in lines 218 – 221 and ethics from 195 to 208.*

**Reviewer #2:**

153 How were the CHWs and CHW leaders recruited for focus groups and interviews?

*We used convenience sampling, selecting participants based on their availability. All CHWs who were part of the study teams were approached, and their team leaders were included as they supervised these CHWs. This has been clarified in the revised manuscript.*

*Our response in lines 185 – 189 are written as follows;*

*“We used convenience sampling for FGDs, with team leaders assisting in identifying available CHWS without disrupting field activities. For IDIs, we included all available team leaders, nurses, and outreach stakeholders from an exhaustive list. We monitored responses during data collection and stopped when no new themes emerged, indicating that saturation had been reached.”*

159 Why was data collection so delayed? Is there a risk that the information collected is reflected on CommCare based mHealth app or that there may be recall bias?

*We recognise this point. The collection of qualitative data in Ekurhuleni experienced delays due to two overlapping disruptions: the district’s transition from AitaHealth to CommCare and the onset of the COVID-19 pandemic. At one stage, it was unclear whether interviews could be conducted at all. We acknowledge that these delays pose a risk of recall bias. However, this bias primarily affects the ability to recall and distinguish between AitaHealth and CommCare, rather than the mHealth experience itself. CHWs continued using the same tablets, and many workflows and functions developed for AitaHealth were retained within CommCare. In this context, we believe CHWs’ reflections remain valid and valuable insights into mHealth implementation under routine conditions. The continued use of these tools, despite the platform switch, highlights the importance of system-level sustainability. CommCare was adopted due to its availability at no cost to the programme through PEPFAR support. We have also added this to the limitations.*

*We have now included the paragraph below in lines 725-732*

*In Ekurhuleni, qualitative data collection continued after CHWs transitioned from AitaHealth to the CommCare platform, which retained many of AitaHealth’s original workflows but was developed and managed by a different provider. Although participants were asked to reflect specifically on AitaHealth, their responses may have been influenced by subsequent use of CommCare. This limitation is worth acknowledging; however, the transition itself may be seen as a positive indicator of adoption and sustainability, as it demonstrates that the core principles and workflows introduced through AitaHealth were preserved and continued on the new platform.*

- Table 4 is confusing.

The way that active days is defined in the methods (the number of days on which CHWs actually used AitaHealth) suggests that “ÁitaHealth Active days” actually be called AitaHealth Outreach days and actual days should be renamed Active days. The list of indicators says % outreach days used as an outcome (which is the proportion of actual days/outreach days) so I would that language in the table (rather than % active days used)

*We agree that our initial definitions were counterintuitive and caused confusion. We have updated the terminology to better align with the more intuitive understanding of the days, following the reviewer’s suggestion. Specifically, active days now refer to days when AitaHealth was made available to CHWs for use in the field, while outreach days refer to days when CHWs actually went out and recorded its use. Since we did not have any other record of CHW attendance, outreach days are only captured through this record on AitaHealth. We have revised the definitions accordingly in the Methods section and updated Table 4 to reflect these changes.*

Table 4: Overall AitaHealth usage statistics

| **District** | **Ekurhuleni** | **uMkhanyakude** |
| --- | --- | --- |
| **Dates when the study was implemented** |  |  |
| *Start date* | 20-Sep-2018 | 18-Jun-2018 |
| *End date* | 02-Sep-2019 | 27-Mar-2019 |
| *Months of implementation* | 11.5 months | 9 months |
| **Day when AitaHealth was available for use** | **347** | **282** |
| *Working weekdays* | 248 | 203 |
| *Weekends* | 99 | 79 |
| **Days when AitaHealth was used by CHWs** | **147** | **111** |
| *Weekday use* | 135 (54.4% of weekdays) | 102 (50.2% of weekdays) |
| *Weekend use* | 12 (12.1% of weekends) | 9 (11.4% of weekends) |
| *% day with any use* | 42.4% (147/347) | 39.4% (111/282) |
| *% of days timestamped use* | 38.3% (133/347) | 34.8% (98/282) |

- Table 6 is also very confusing

Is the Completed household assessments column with n (%) the number of households where this question was completed (and proportion)?

*The ‘Completed household assessments’ column was intended to show data completeness, representing the number and percentage of households with the relevant field filled in, regardless of response. The ‘Triaged participants’ column, meanwhile, indicates outcomes, showing the number and percentage of households where at least one member actually met the specified condition. We recognise that this distinction was not fully clear in the original version. Therefore, we have revised the footnotes to define both terms explicitly and updated the Results text to clarify these two aspects. We hope this clarification resolves the ambiguity and makes Table 6 easier to interpret.*

Table 6 : Data completeness and triaged participants

| **Variables** | **Completed household assessments** | | **Triaged participants** | |
| --- | --- | --- | --- | --- |
|  | **n** | **%** | **n** | **%** |
| **Household characteristics (n=2452)** |  |  |  |  |
| *Household triaged* | 1945 | *79.3%* |  |  |
| *Has GPS coordinates* | 1866 | *76.1%* |  |  |
| **Tuberculosis indicators (n=2129)** |  |  |  |  |
| *≥1 member currently on TB treatment* | 2049 | *96.2%* | 211 | *8.6%* |
| *≥1 member completed TB treatment (past 12 months)* | 2045 | *96.1%* | 123 | *5.0%* |
| *≥1 member diagnosed, not on treatment (past 6 months)* | 2043 | *96.0%* | 68 | *2.8%* |
| *≥1 member defaulted after TB diagnosis* | 2043 | *96.0%* | 92 | *3.7%* |
| *≥1 member had TB symptoms* | 2016 | *94.7%* | 152 | *6.2%* |
| **HIV, Home-based care & Maternal Health (n=2129)** |  |  |  |  |
| *≥1 member required home-based care* | 1998 | *93.9%* | 76 | *3.1%* |
| *≥1 member HIV-exposed* | 1998 | *93.9%* | - | - |
| *HIV test requested for more than one member* | 2027 | *95.2%* | 238 | *9.6%* |
| *≥1 pregnant member (ANC)* | 2012 | *94.5%* | 142 | *5.8%* |
| *≥1 member receiving post-natal care* | 2001 | *94.0%* | 78 | *3.2%* |
|  | | | | |
| *^1^ Completed household assessments = number (and %) of households where the relevant field was filled in the tool, regardless of the response entered.* | | | | |
| *^2^ number (and %) of households where at least one member actually met the specified indicator (e.g., on TB treatment, reported TB symptoms)* | | | | |

Why is it if the tool is used for TB contact tracing that not 100% of households had a member diagnosed with TB?

*AitaHealth was developed as a multipurpose digital tool to support CHWs in household outreach, addressing TB, HIV, maternal and child health, and other services. TB contact tracing was one of its features, but the tool was not restricted to households with a TB diagnosis. Instead, it was widely used for household screening, referrals, and follow-up across multiple conditions. We deliberately adopted this approach because focusing solely on TB contact tracing would have overlooked broader household needs, and we believe that standalone TB-specific tools are unlikely to succeed in practice, given the multiple demands faced by CHWs. Additionally, CHWs did not only target the homes of household contacts of people being treated for TB, so limiting the tool to TB alone would have restricted its usefulness, especially in areas without TB patients. Having a universal tool also enables indiscriminate screening for TB without missing cases, which would not have been possible if they had continued using paper.*

- Given the problems with battery life that was mentioned, is there a Is there some way to determine how many days the AitaHealth system actually accessible to use in the field? For example, health worker goes with the tablet but then tablet dies, it may be inaccessible for half the day (or is this what was measured in active days?). This would help strengthen the conclusion that mHealth app’s limitations were primarily due to system constraints rather than user resistance.

*These were indeed the active days. As clarified in the revised definitions, and consistent with the reviewer’s phrasing, active days now refer to days when the tool was accessible for use in the field. The difference between active days and actual use reflects periods of non-access, but we cannot attribute this gap to specific causes, as reasons were not tracked systematically.*

468 Was it ever explored why the app was used less on the weekends? This seems to be pointing to a user preference issue rather than a technology issue if there’s such marked difference between use on weekdays and weekends.

*We did not explore this in detail because CHWs were not expected to work on weekends. What surprised us during the analysis was that some of them actually decided to work on weekends. This is the same group that had raised concerns about how they were remunerated, so to see them go the extra mile outside of regular workdays. We do not believe the weekend usage pattern was related to technology, as there is no indication that the tool or devices functioned differently on weekends. The more likely explanation is their commitment to the work. We consider this an incidental but important finding, and we have now reflected it in the manuscript.*

482 Should the number of entries been a median rather than a mean?

*We agree that medians are more suitable here and have now updated the analysis accordingly. We also realised that the original figures for this specific row included entries without timestamps, which we now report in Table 5. In doing so, we have also removed the confusing footnote, which somewhat implied that some CHWs were not working.*

Table 5: Overall household and data entry summary

| **Metric** | **Ekurhuleni** | **uMkhanyakude** | **Total** |
| --- | --- | --- | --- |
| **A. CHW Deployment** |  |  |  |
| *Total CHWs* | 61 | 42 | 103 |
| **B. Data Captured in AitaHealth** |  |  |  |
| *Total entries recorded* | 5,096 | 5,553 | 10,649 |
| *Entries with timestamps (%)* | 4,677 of 5,096 (91.8%) | 4,213 of 5,553 (75.9%) | 8,890 of 10,649 (83.6%) |
| *Households recorded* | 1,514 | 938 | 2,452 |
| *Household size,* mean; p50, (sd) | 4.3; 4, (2.4) | 7.9; 7, (4.0) | 6.2; 6, (3.8) |
| *Households per CHW,* mean; p50, (sd) | 24.8; 18, (25.7) | 22.3; 19.5, (16.4) | 23.8; 18.2, (22.3) |
| *Timestamped entries per CHW,* mean; p50, (sd) | 76.7; 60, (68.2) | 100.3; 48.5, (118.7) | 86.3; 57.9, (92.4) |
| *Timestamped entries per outreach day,* mean; p50, (sd) | 35.2; 26 (38) | 43; 26, (43.1) | 38.5; 26, (40.3) |
|  |  |  |  |
| **C. CHW Login Activity** *(61 in Ekurhuleni, 42 in uMkhanyakude)* |  |  |  |
| *CHWs with 75–136 logins* | 5 (8.2%) | 5 (11.9%) | 10 (9.7%) |
| *CHWs with 30–74 logins* | 10 (16.4%) | 8 (19.0%) | 18 (17.5%) |
| *CHWs with 15–29 logins* | 15 (24.6%) | 10 (23.8%) | 25 (24.3%) |
| *CHWs with 5–14 logins* | 16 (26.2%) | 12 (28.6%) | 28 (27.2%) |
| *CHWs with 1–4 logins* | 15 (24.6%) | 7 (16.7%) | 22 (21.4%) |
|  | | | |

494 94% of households had TB fields completed - is that any entry or each individual logged in the household also had questions asked

*The 94% refers to households where at least one TB-related field was completed in the digital tool. This does not mean that every individual household member had a full TB assessment recorded. This can also be understood in relation to the earlier comment about Table 6, where we made several changes to improve the table’s clarity.*

590 “CHWs incorporated AitaHealth into their outreach but used it inconsistently, not because of poor design or user resistance but because the conditions needed for sustained engagement were not in place” I don’t think that this conclusion can be made without understanding how limiting the technological barriers were

*Thank you for raising this point. We agree that technological barriers were a significant part of the broader system constraints that influenced the inconsistent use of AitaHealth. In our setting, it was challenging to quantify the specific impact of individual barriers, such as battery failure, freezing, or app malfunctions, because these were not systematically monitored during implementation. This is a recognised limitation of this pragmatic evaluation. However, we understood the limitations of these technological barriers through repeated and specific descriptions provided during qualitative interviews with CHWs across both provinces. These issues were independently raised by CHWs working in different teams, interviewed months apart, and with no interaction or chance to influence one another. The fact that the same challenges emerged under these conditions shows they were common experiences across settings. CHWs consistently described how technology failures disrupted their work and affected their ability to use the tool regularly. These accounts were not isolated and were provided alongside discussions of other systemic issues, such as lack of support, safety concerns, and frustration with working conditions. Collectively, these narratives give us a reasonable basis to understand the nature and impact of technological barriers, even if we cannot assign them a numerical weight. We have revised the manuscript to clarify that our conclusion is based on this triangulated evidence. The statement is not intended to exclude or minimise technological issues, but to show that inconsistent use was shaped by multiple overlapping constraints, technical, operational, and environmental, rather than poor tool design or lack of willingness among CHWs. As an additional measure and in response to further comments from a different reviewer, we conducted an exploratory mixed-effects model to better understand some of these dynamics. The results of this model are in the appendices.*

*The responses to this are spread across the discussion, particularly from lines 607 downwards to line 634. We provide the revised discussion here;*

*Feasibility challenges in our study reflect patterns observed elsewhere, where digital tools are often well-received by CHWs but remain difficult to use consistently in routine conditions {Guzman, 2023 #505}. Although South Africa’s CHW programme and infrastructure differ from those in Uganda, Tanzania, and Malawi, similar challenges, including poor connectivity, device failures, and stigma, despite user acceptability, reinforce the broader relevance of our findings {Tumuhimbise, 2023 #499;Kachimanga, 2024 #504;Mtenga, 2024 #571}. This disconnect is well established in implementation science, where acceptability alone does not guarantee adoption {Proctor, 2011 #497;Proctor, 2023 #496}, a point clearly articulated by Dimanopoulos and colleagues, who note that “acceptability may predict adoption, [but] it does not guarantee engagement”, particularly when contextual and structural barriers persist {Dimanopoulos, 2025 #568}. Broader implementation frameworks also caution that adoption unfolds gradually, requiring time, policy alignment, and sustained infrastructural support to become embedded in daily workflows {Lennon, 2017 #570;World Health Organization, 2019 #416}. It is therefore plausible that our evaluation, like others, captured an early phase of implementation before adoption had stabilised. Nonetheless, these challenges are no longer unforeseen; future mHealth programmes must anticipate them and proactively plan to mitigate their effects.*

*Device quality was a recurring challenge, with CHWs often experiencing battery failures, overheating, and fragile hardware that undermined consistent usage. In our study, devices degraded rapidly, likely reflecting their overall quality and the demands of daily fieldwork. For many CHWs, this was their first time using such a device. Although we did not track non-study usage, they could also have used them for personal purposes, which would have added to the wear and tear. These experiences emphasise the need for structured device management, including regular inventories, replacement plans, and accountability systems. However, approaches that entirely restrict personal use may be counterproductive, as CHWs valued these devices and incorporated them into their daily routines. A more effective approach, we believe, is to provide durable equipment and establish systems to manage and monitor them responsibly, while maintaining CHWs’ motivation to use digital tools in their work.*

608 What is the impact of the technology on the TB program? Was there a clinical or reporting improvement due to the technology? What was the number or proportion of referrals and did this change after implementation? Was there a change in the timeliness of reporting or the fidelity of the information compared to paper records? Were there more households screened overall?

*As noted in the discussion, we were unable to reliably link CHW field activities to clinic-level outcomes, such as TB diagnosis or completion of referrals. Firstly, we simply nested some TB contact tracing within a large outreach programme and only did so for one year, a period that we believe may be too short, allowing us to measure only early implementation outcomes. Patients were free to attend any facility, and the available statistics could not be specifically attributed to AitaHealth use. Our study was designed as an implementation evaluation on a limited scale, with the primary aim of testing whether digital contact tracing was acceptable, feasible, and usable by CHWs under routine conditions. Measuring programme impact requires a different design, such as a randomised trial or large-scale implementation like ZAMSTAR, where district- or province-wide outcomes can be assessed. We therefore see our contribution as providing the foundational evidence needed before claims about population-level impact can be made.*

*We provide the explanation below in lines 708-721*

*We also struggled to link household contact tracing data to clinic-level outcomes, such as TB diagnosis. While facility statistics were available, attributing outcomes to AitaHealth use was challenging because follow-up actions often rely on other personal and clinic factors beyond the control of CHWs. This reflects a broader issue with many interventions, including some mHealth, which can enhance data quality and efficiency, but their impact on clinical outcomes may be indirect unless systematic support for the entire cascade {Hanrahan, 2019 #387}. Measuring programmatic impact would have required a larger design implemented for longer, such as ZAMSTAR, a cluster-randomised trial in Zambia and South Africa that evaluated community-wide household interventions with population-level outcomes {Ayles, 2008 #310}. In contrast, our study was designed to generate foundational evidence on feasibility, acceptability, and fidelity under routine conditions, as a necessary precursor to larger-scale trials or programs capable of assessing epidemiological impact. More integrated approaches connecting CHWs, clients, and facilities may be needed to bridge this gap. This scale and length limitation also meant that we were unable to conduct a formal validation of data accuracy by comparing digital entries to paper-based clinic records due to resource and access constraints.*

Was there a change in usage over the timeframe of the study which may have reflected battery life etc?

*In our study, we observed that the devices themselves degraded over time, with battery failures, overheating, and fragility becoming increasingly common. This likely reflected both their modest specifications and the intensity of daily fieldwork, with possible additional wear from personal use, given that for many CHWs it was their first time receiving such a device. As we noted in the discussion, these experiences highlight the need for more durable equipment and structured device management, rather than restrictive measures that could disengage CHWs from tools they value. Some of this is covered in the mixed-effects model that we now provide.*

*We now have a paragraph from lines 623 – 634*

*Device quality was a recurring challenge, with CHWs often experiencing battery failures, overheating, and fragile hardware that undermined consistent usage. In our study, devices degraded rapidly, likely reflecting their overall quality and the demands of daily fieldwork. For many CHWs, this was their first time using such a device, and although we did not track non-study usage, they could also have used them for personal purposes, adding to the wear and tear. These experiences emphasise the need for structured device management, including regular inventories, replacement plans, and accountability systems. However, approaches that entirely restrict personal use may be counterproductive, as CHWs valued these devices and incorporated them into their daily routines. A more effective approach, we believe, is to provide durable equipment and establish systems to manage and monitor them responsibly, while maintaining CHWs’ motivation to use digital tools in their work.*

494 When you say “Over 94% of the 495 2,452 households had TB-related fields completed, including treatment status, 496 symptom screening, and treatment history” do you mean every member of the household had this information filled in or at least one member had this question asked?

*We have corrected the statement, as well as others in the same paragraph, to match the table. The table footnotes had already noted that this was at the household level, but the text did not reflect this consistently. The revised Results paragraph now explicitly states that outcomes refer to at least one member in the household.*

**Reviewer #3:**

 Overview

This mixed-methods study evaluates AitaHealth implementation for TB contact tracing by community health workers in two South African districts. The manuscript provides valuable insights into real-world mHealth implementation challenges.

However the research questions and sampling strategy need clearer articulation. Additionally, integration between qualitative and quantitative findings could be strengthened throughout. The apparent contradiction between high acceptability and low usage rates requires better explanation. Several outcomes, including device failure rates and safety incidents, lack quantification.

Introduction
● Lines 54-56: The statement “20% were not reached” is ambiguous. Please specify what this gap refers to.

*Thanks for highlighting this. We have revised the sentence to make it clear that the 20% refers to people with TB who were never reached by testing or treatment services. We also added that this leads to missed diagnoses and contributes to further inefficiencies downstream, including poor care linkage and fewer chances for TB preventive therapy (TPT) among household contacts.*

*The revised sentence is line 55-58 reads as follow;*

*However, around 20% were never reached by testing or treatment services, resulting in missed diagnoses and downstream inefficiencies, including poor linkage to care and fewer opportunities for TB preventive therapy (TPT) among household contacts {Velen, 2016 #384;Page-Shipp, 2018 #443;Kaswaswa, 2022 #490;Hamada, 2024 #427}.*

● Lines 69-70: The reference to the Ugandan mHealth trial notes that it “failed to improve outcomes.” Please specify which key outcomes the strategy failed to improve

*We have revised the sentence to specify the outcomes. The updated text now makes it clear that the mHealth strategy did not improve either the completion of TB evaluation within 14 days or the detection of new TB cases compared to standard clinic-based contact investigation. This provides a clearer understanding of the intervention’s limitations and strengthens the rationale for context-specific implementation evaluations.*

*Lines 72 – 76 now read as follows;*

*A trial in Uganda illustrated this; it was only through a detailed process evaluation that researchers were able to understand why the mHealth strategy failed to increase the proportion of household contacts who completed TB evaluation within 14 days, and why it did not improve case detection compared to standard clinic-based contact investigation {Meyer, 2018 #229;Gupta, 2023 #436}.*

● Lines 60-61: The introduction identifies the challenges of paper-based systems and then introduces mHealth as a potential solution. You could make this link even more explicit by stating directly how mHealth features (e.g., automated reminders, digital data capture) are theorised to address the specific problems of “delays, data loss, and missed follow-ups” that you mention.

*We have revised the sentence to clearly connect the challenges of paper-based systems with the features of mHealth tools designed to address them. Specifically, we added a reference to “by enabling quicker data capturing, automated reminders, defined workflows and real-time monitoring and reporting” to demonstrate how these tools aim to reduce delays, data loss, and missed follow-ups.*

*The revised content is now in `lines 64-68 reading as follow;*

*Although mobile health (mHealth) tools like Mobenzi and CommCare can ease routine tasks by enabling quicker data capturing, automated reminders, defined workflows and real-time monitoring and reporting {Neupane, 2014 #453;Maraba, 2018 #120;Wood, 2019 #111;Lindberg, 2019 #480;Neupane, 2014 #453;Nelson, 2020 #439;Tshikomana, 2022 #452}, their impact on TB outcomes remains unclear {Odendaal, 2020 #206;Ojo, 2018 #506}, and integration into the health system is limited {Van der Pol, 2021 #195}.*

● Lines 73-77: The introduction states that the study ‘evaluates the tool’s acceptability, feasibility, and implementation fidelity’ but doesn’t present specific research questions. Consider adding explicit research questions e.g. acceptable to whom?

*We have revised the text to state the research questions guiding our evaluation explicitly. The updated section now also clarifies that although AitaHealth was the specific platform used, the study aimed to inform broader mHealth implementation in TB contact tracing, as previously asked. We now explicitly frame the evaluation around three core questions: acceptability, feasibility, and fidelity, as applied to CHWs, team leaders, and healthcare workers in routine outreach.*

*We now have these in lines 85-88 reading as follows;*

*Specifically, we asked: (i) was the platform acceptable to CHWs, team leaders, and healthcare workers? (ii) was it feasible for CHWs to use during routine contact tracing? and (iii) was the tool implemented as intended in real-world outreach?*

Methods
● The methods don’t explain how the two districts were selected or describe the sampling strategy. How did you recruit participants? How did you know when you reached a sufficient sample size?

*We have revised both the Study Background and Setting, as well as the Data Collection sections, to address this. The paragraph on district selection has been restructured to follow the comparison of settings, and now clearly explains that we selected the two districts based on their contrasting urban–rural contexts, high TB and HIV burdens, and the fact that our teams were already active in both locations with established WBOT platforms.*

*Lines 106 – 109 introduce the following;*

*We selected these two districts because they offered contrasting implementation contexts: one rural and one urban, and because our teams were already active in both. Their high TB and HIV burdens, along with well-established CHW outreach systems, made them ideal for testing an mHealth tool under real-world conditions.*

*We also clarified our sampling strategy. For focus group discussions, we used convenience sampling, with CHW team leaders helping identify participants available on the day of data collection to minimise disruption to fieldwork. For in-depth interviews, we used an exhaustive approach, inviting all relevant outreach staff and stakeholders involved in programme oversight in each district. We monitored responses during data collection and concluded interviews once no new themes emerged, indicating that thematic saturation had been reached.*

*The sampling revision is in lines 185 – 189*

*We used convenience sampling for FGDs, with team leaders assisting in identifying available CHWS without disrupting field activities. For IDIs, we included all available team leaders, nurses, and outreach stakeholders from an exhaustive list. We monitored responses during data collection and stopped when no new themes emerged, indicating that saturation had been reached.*

● The training description is brief. Please provide more detail on the duration of training sessions, whether training was standardised across sites, any competency assessments conducted, and whether refresher training was provided during the implementation period.

*We have expanded the Methods section to cover all the points raised. We now clarify that training was standardised across all sites, conducted in person before implementation, included a competency check using dummy data entry, and was delivered jointly by developers. Although no formal refresher training was provided, CHWs received remote support as needed.*

*We have now revise the training section with the recommendations in lines 150 – 159*

*Before implementation, CHWs in uMkhanyakude and Ekurhuleni received a once-off, in-person AitaHealth training session in their respective districts delivered by the developers. Training was standardised across sites and carried out shortly before rollout. It covered how to use AitaHealth, including navigation, entering household-level data, and operating it during outreach. To assess competency, CHWs completed practical tasks of capturing dummy data directly into AitaHealth during the session. It also included basic TB management, contact tracing, and TB preventive treatment, led by facilitators from the University of Pretoria’s Department of Family Medicine. Although no formal refresher training was provided during the implementation period for using AitaHealth, CHWs received ongoing remote technical support as needed.*

● The manuscript mentions that the Ekurhuleni district transitioned from AitaHealth to the CommCare app in 2021 (line 142). Since qualitative data collection in Ekurhuleni continued until May 2022, it is possible that CHWs’ experiences with CommCare influenced their reflections on AitaHealth. The authors should acknowledge this in the limitations.

*We thank the reviewer for highlighting this important point. In Ekurhuleni, some qualitative data were collected after CHWs had transitioned from AitaHealth to the CommCare platform, which retained many of AitaHealth’s original workflows. Although participants were asked to reflect specifically on AitaHealth, their experiences might have been influenced by subsequent exposure to CommCare. We have now acknowledged this as a limitation in the manuscript, noting that it could have affected the findings on usability and acceptability. This point was also raised by another reviewer regarding delayed data collection and recall bias, and we have aligned our responses so that both issues are addressed consistently.*

*We have now included the paragraph below in lines 725-732.*

*In Ekurhuleni, qualitative data collection continued after CHWs transitioned from AitaHealth to the CommCare platform, which retained many of AitaHealth’s original workflows but was developed and managed by a different provider. Although participants were asked to reflect specifically on AitaHealth, their responses may have been influenced by subsequent use of CommCare. This limitation is worth acknowledging; however, the transition itself may be seen as a positive indicator of adoption and sustainability, as it demonstrates that the core principles and workflows introduced through AitaHealth were preserved and continued on the new platform.*

● Line 88: The phrase ‘TB has also been noted as the leading cause of disease’ is imprecise. Please clarify whether this refers to death, cases or morbidity. And during what year or time period?

*We thank the reviewer for flagging this. We revised the phrasing to mention mortality specifically and included the relevant period. We now state that, in uMkhanyakude, TB is the second leading cause of death after HIV, based on death-notification data from 2012 to 2017 reported in the District Health Barometer and confirmed by the 2023/24 Integrated Development Plan.*

*We now write it as follows in lines 99-102*

*In uMkhanyakude, TB was the second-leading cause of death after HIV, according to mortality data from 2012–2017 as reported in the 2023/24 Integrated Development Plan and District Health Barometer {Municipality, 2023 #509;Massyn, 2020 #567}.*

● Lines 92-94: The final sentence comparing HIV prevalence between districts is awkward and unclear. Consider revising

*We revised this section to improve clarity and structure. Instead of combining everything into a single paragraph, we now describe each district in its own brief paragraph to better highlight the differences between settings rural versus urban, population density, poverty levels, and disease burden. The previously unclear sentence has been rephrased and incorporated into the broader comparison, rather than standing alone. This revision also addresses an earlier comment about district selection. We added a short final paragraph explaining why these two districts were chosen.*

● Lines 108-115: While technical specifications are provided for the study tablets, it is unclear whether AitaHealth can be deployed on commercially available devices (e.g., iPads, Samsung tablets) or requires specific hardware.

*Thank you. We have clarified that the tablet used in the study was commercially available and that AitaHealth runs on standard Android devices. It is not compatible with Apple’s iPhone operating system.*

*We now include the following in lines 133- 134*

*AitaHealth is designed for Android devices and is not compatible with Apple’s iPhone operating system (iOS).*

Results
● Table 2 shows the number of participants but lacks demographic information (age, gender, years of experience, education level) that could help contextualise the findings, particularly given comments about older CHWs struggling with technology. Is it possible to include this detail?

*We have added summary demographic data (age and years of service) for CHWs. These additions help contextualise the findings, especially regarding user experience and digital familiarity. We only collected this information for CHWs who participated in Focus Group Discussions.*

Table 2: Summary of Qualitative Data Collection

| **Category** | **Ekurhuleni** | **uMkhanyakude** | **Total** |
| --- | --- | --- | --- |
| Data collection period | 2019–2022 | 2021 |  |
| *Age of FGD participants (years),* mean; p50, (±sd) | 41.8; 37.5 (±9.4) | 44.7; 45, (±7.4) | 43.3; 42.5, (8.9) |
| Years of service | *10.3; 11 (±0.9) | *7.6; 7, (±4.4) | 8.9; 10, (3.4) |
| **Focus Group Discussions (FGDs)** | **2** | **2** | **4** |
| - ^1^*CHWs in FGDs* | *25* | *43* | *68* |
| **In-Depth Interviews (IDIs) (Total)** | **6** | **4** | **10** |
| *- Tuberculosis Nurses in IDIs* | *2* | *2* | *4* |
| *- CHW Team Leaders in IDIs* | *2* | *2* | *4* |
| *- Key Stakeholders in IDIs* | *2* | *0* | *2* |
| *^1^ CHWs -Community Health Workers*  ** p ≤ 0.05*  *Data were collected in uMkhanyakude (2021) and in Ekurhuleni (IDIs 2019–2021; FGDs 2022)* | | | |

● The qualitative quotes lack temporal indicators. Given that data collection spanned different periods (especially in Ekurhuleni from 2019-2022), it would be helpful if you indicated when each quote was collected, as perceptions may have evolved over time.

*We thank the reviewer for this helpful suggestion. To address it, we have added temporal information to Table 2 to indicate when the qualitative data were collected. In KZN, both FGDs and IDIs were conducted over two days. In Ekurhuleni, FGDs were collected in 2022, while IDIs and other interviews took place between 2019 and 2021. Because mHealth tools continued to be used during this period, we believe this summary in Table 2 provides the necessary context for interpreting the quotes, rather than adding dates to each quote individually.*

● Table 5 suggests nearly half of CHWs logged in fewer than 15 times over 9-12 months. This seems inconsistent with claims of acceptability. Could you please explain this? Is the result of the specific barriers mentioned in qualitative findings? Consider adding a section in the results where you integrate the quantitative and qualitative findings.

*We agree that the observed gap between low login frequency and high reported acceptability warrants clarification. This pattern reflects a well-documented distinction in implementation science: that perceived acceptability does not necessarily lead to sustained adoption. In our response, however, we found that adding an integrated qualitative and quantitative section to the results was duplicative, so we chose to incorporate them more analytically within the discussion. We hope this is acceptable. The discussion now elaborates on this disconnect with references. This revised section also combines a response to a later comment about comparing different countries with varying contexts.*

● The description of hardware and software failures is concerning but lacks quantification. What proportion of devices failed? How many CHWs reverted to paper?

*Thank you for this important observation. Hardware and software issues were reported primarily through qualitative interviews. Unfortunately, we did not have a systematic mechanism in place to quantify device failures or track the number of community health workers who reverted to using paper. This is a limitation of the study, which we have now acknowledged in the discussion under the limitations section.*

● CHWs expressed significant safety concerns, but it’s unclear if any actual incidents occurred. Please clarify whether any devices were stolen or CHWs were harmed

*This is a valuable and much-appreciated comment. We recognise that we could have proactively tracked or documented incidents such as theft, device damage, or threats to CHW safety more systematically during implementation. However, the study was conducted as a pragmatic project, where our role was limited to providing the digital tool and technical support, not overseeing day-to-day operational activities led by district teams. As a result, we did not collect certain operational details that, in hindsight, would have been beneficial. This is an important lesson. We have now clarified the scope of our involvement in the revised Methods section, and we address this gap both in the discussion (as a programmatic consideration) and under Limitations (as a missed opportunity for more structured tracking and mitigation).*

*Lines 643 -645 address this;*

*Although no incidents of theft or harm were formally reported to the study team, we acknowledge that we could have played a more proactive role in tracking such events. This was a missed opportunity and represents a key limitation in capturing risks associated with outreach fieldwork.*

● While data is presented for both sites, there’s limited comparative analysis. Given the different contexts (rural vs urban), what differences emerged in implementation experiences between sites?

*We thank the reviewer for this helpful comment. We have expanded the Results and Discussion to compare the two districts more clearly. In uMkhanyakude, CHWs demonstrated stronger early uptake and enthusiasm, despite more challenging conditions, while in Ekurhuleni, engagement was lower, but digital tools persisted post-study through programme-level adoption. We now highlight this contrast to illustrate how micro-level uptake can struggle without structural support, whereas macro-level backing can sustain use even with lower initial engagement. Our exploratory mixed-effects models, which we have in the appendices, also partially support this.*

*Lines 680-694 address this as follows;*

*Implementation experiences varied between Ekurhuleni (urban) and uMkhanyakude (rural), demonstrating how local context and team dynamics shape digital engagement. CHWs in uMkhanyakude used AitaHealth more often, despite facing weaker infrastructure and more challenging working conditions. This was unexpected, as higher usage was expected in the urban setting. Our mixed-effects model indicated that these differences might have been influenced by unmeasured team-level factors, such as supervision quality or informal leadership, rather than solely by individual or household traits. However, this increased use did not determine sustainability. AitaHealth did not continue in either district; it was discontinued in uMkhanyakude due to lack of funding, and in Ekurhuleni, it was replaced with a free transition to CommCare, highlighting the absence of long-term financial planning as a central barrier to sustainability. To attract future investment, programmes need to demonstrate not only uptake but also generate evidence aligning with funders’ priorities. This includes not only usage data but also economic evidence to inform decisions on resource use and allocation.*

● There’s no assessment of data accuracy. Were any validation checks performed to ensure the quality of entered data compared to paper records?

*We recognise this important point. Due to resource limitations and the pragmatic design of our study, we did not conduct clinic-level validation to compare AitaHealth entries with paper records. Our primary objective was to evaluate the feasibility and usability of the tool during routine outreach. We agree, however, that validating digital data against facility-based records is an important next step, and we have added this as a recommendation in the discussion.*

Discussion
● The opening paragraph summarises findings but there appears to be a contraindication between ‘well accepted’ and usage on ‘fewer than half of outreach days.’ In the discussion of fidelity it is suggested that this is driven by system level factors (battery, wifi). Could low usage also reflect rational CHW decisions about when digital tools add value versus create burden?

*We thank the reviewer for this thoughtful observation. We have revised the opening of the discussion to address the apparent tension between high acceptability and lower usage. Our data show that CHWs did not consistently use AitaHealth across all outreach days, but this does not mean the tool was poorly accepted. CHWs described it as useful and aligned with their work, yet usage was constrained by factors such as unreliable devices, weak connectivity, and safety concerns. We cannot determine from our data when CHWs chose to revert to paper, only that the tool was not used all the time. We have clarified this point in the discussion and expanded on it later in the section by drawing on implementation research, which shows that outcomes such as acceptability, feasibility, and fidelity are interrelated but do not always directly lead to one another.*

*569 – 580 (opening paragraph of the discussion)*

*Discussion*

*AitaHealth was well-received by CHWs when it was operational and was perceived to improve efficiency, data accuracy, and referral processes. Supervisors and stakeholders valued its real-time monitoring capabilities and automated reporting functions. Feasibility, however, was constrained by unreliable hardware, limited technical support, and insecure working conditions, which directly affected implementation fidelity. Despite the high acceptability evident in qualitative interviews, the tool was used on fewer than half of the active days. We interpret this dissonance as a pragmatic, task-level choice where CHWs used the tool when it added clear value and reverted to paper when it created challenges that disrupted their work. This illustrates that even highly acceptable tools will not be used consistently unless operational conditions enable ease of use.*

● While comparing findings to Uganda and Malawi studies, the discussion doesn’t address whether these contexts are sufficiently similar for meaningful comparison. Please acknowledge relevant contextual differences.

*We have revised the discussion to acknowledge that, while South Africa differs from Uganda and Malawi, the feasibility challenges we observed, particularly hardware and support limitations, are echoed across these settings. This consistency strengthens our argument that feasibility is a fundamental determinant of mHealth success, even in diverse contexts.*

*Lines 613 – 616 read as follows;*

*Although South Africa’s CHW programme and infrastructure differ from those in Uganda, Tanzania, and Malawi, similar challenges, including poor connectivity, device failures, and stigma, despite user acceptability, reinforce the broader relevance of our findings [10, 40, 43].*

● The results included perspectives from supervisors and stakeholders, but these voices are largely absent from the discussion. How do supervisor views align or conflict with CHW experiences?

*We have added a paragraph to the discussion addressing this. Supervisors largely supported AitaHealth for its reporting and oversight benefits, even though they acknowledged feasibility issues raised by CHWs. Their stronger buy-in may explain the continued use in Ekurhuleni, emphasising how different user roles influence perceptions of value and sustainability.*

*Lines 695 – 706*

*Supervisors shared many concerns raised by CHWs but viewed them from a broader programme perspective, whereas CHWs mainly judged the tool on its performance in the field. Although we cannot assess the extent to which their views influenced the continuation of implementing mHealth in Ekurhuleni, securing supervisor buy-in is crucial for the adoption and scale-up of these initiatives. Research indicates that middle managers play an important role in translating adoption decisions into practice by coordinating resources, motivating staff, and monitoring progress [48]. Recent South African studies highlight gaps in leadership readiness that could hinder implementation [49]. In our study, Ekurhuleni continued implementing mHealth tools as long as funding was available, but the project stopped in uMkhanyakude once the funding ended. These patterns suggest that funding enables continuation, but sustainable use relies on managerial support.*

● What can be learned from the high frequency users? Did their success reveal strategies that could inform future implementations?

*We appreciate this suggestion and agree that it raises an important point, one we began exploring during analysis after noticing significant variation in usage. Although our study was not specifically designed to identify what drives high usage, and we lack key variables such as supervision intensity, we conducted an exploratory mixed-effects model using the available data. We treated the team the CHWs belonged to as a random effect to account for unobserved differences in supervision and team dynamics and included the district as a fixed effect since only two districts were involved, allowing for direct comparison. We also adjusted for each CHW’s start date, usage span, and number of visits. This model enabled us to examine how usage clusters across individuals, teams, and districts, although we interpret the results with caution. The model output is presented in the Appendix and referenced in the Results section. Future studies should consider collecting structural variables like these from the outset to better understand what influences consistent use.*

*We have attached the model as an appendix as it could be too long to include in the rebuttal. The document is named “****Asibambisane Mixed Effects model Appendix****”*

● The discussion doesn’t address long-term sustainability. What happened after the study ended? The mention of Ekurhuleni switching to CommCare suggests AitaHealth wasn’t sustained - why?

*We appreciate the reviewer’s interest in post-study sustainability. In uMkhanyakude, to our knowledge, AitaHealth was not sustained after the study, and the district reverted to paper-based data collection. There was no funding available to continue using AitaHealth. In Ekurhuleni, AitaHealth was also not sustained, but mHealth activities continued because CommCare was offered to the district free of charge by a PEPFAR partner. AitaHealth continued as a tool operated by the University of Pretoria but appears to have been later acquired by Vodacom and is now commercialised through their subsidiary Mezzanine Ware. These examples are now incorporated into the discussion, which highlights that even when tools are used enthusiastically, long-term continuation depends on whether a payer is present. Our study demonstrated that AitaHealth was feasible, acceptable, and aligned with implementation goals; however, long-term sustainability requires a structural and financial commitment from either the government or a donor.*

*Lines 688 – 694 address the comment as follows;*

*However, this increased use did not determine sustainability. AitaHealth did not continue in either district; it was discontinued in uMkhanyakude due to lack of funding, and in Ekurhuleni, it was replaced with a free transition to CommCare, highlighting the absence of long-term financial planning as a central barrier to sustainability. To attract future investment, programmes need to demonstrate not only uptake but also generate evidence aligning with funders’ priorities. This includes not only usage data but also economic evidence to inform decisions on resource use and allocation.*

● The discussion doesn’t engage with the economics of mHealth implementation. What are the cost implications of providing devices, support, and training versus potential efficiency gains?

*We thank the reviewer for highlighting this crucial point. Although a detailed economic analysis was outside the scope of this paper, we agree that cost considerations are central to adoption and long-term sustainability. We have now addressed this in the discussion, alongside our comments on sustainability, noting that while AitaHealth was actively used in some settings, it was not continued after the study due to a lack of funding, and that long-term use depends on a willing payer. We acknowledge that broader economic evidence is needed to inform whether, how, and where mHealth tools should be funded and scaled.*

*Lines 691 – 694 respond as follow;*

*To attract future investment, programmes need to demonstrate not only uptake but also generate evidence aligning with funders’ priorities. This includes not only usage data but also economic evidence to inform decisions on resource use and allocation.*

6. PLOS authors have the option to publish the peer review history of their article ([what does this mean?](https://journals.plos.org/globalpublichealth/s/editorial-and-peer-review-process#loc-peer-review-history)). If published, this will include your full peer review and any attached files.

**Do you want your identity to be public for this peer review?** If you choose “no”, your identity will remain anonymous but your review may still be made public.

*No*

Yours sincerely,

Don Mudzengi

Email:	[dmudzengi@auruminstitute.org](mailto:dmudzengi@auruminstitute.org)

Cellphone:	+27 (0) 83 369 7946

Landline:	+27 (0) 10 590 1388
