# Supplementary material for: Evaluating the acceptability, feasibility, and implementation fidelity of a multipurpose mobile health (mHealth) app used for Tuberculosis (TB) contact tracing by Ward-Based Outreach Teams (WBOTs) in South Africa
Source: PLOS Glob Public Health. 2026 Aug 31;6(8):e0007120. doi: 10.1371/journal.pgph.0007120 (PMC13528960; doi:10.1371/journal.pgph.0007120)
Supplement: Peer review history

# Peer review: Decision Letter 0

**DOI:** 10.1371/journal.pgph.0007120.r001

**Authors:** Suman Majumdar

29 Jul 2025

PGPH-D-25-01506

Evaluating the acceptability, feasibility, and implementation fidelity of a multipurpose mobile health (mHealth) app used for Tuberculosis (TB) contact tracing by Ward-Based Outreach Teams (WBOTs) in South Africa

PLOS Global Public Health

Dear Dr. Mudzengi,

Thank you for submitting your manuscript to PLOS Global Public Health. After careful consideration, we feel that it has merit but does not fully meet PLOS Global Public Health’s publication criteria as it currently stands. Therefore, we invite you to submit a revised version of the manuscript that addresses the points raised during the review process.

Kindly address all reviewer comments.  In particular, reviewer 2 and 3 have made sound suggestions to strengthen the presentation of the results and the discussion.

Please submit your revised manuscript by Sep 12 2025 11:59PM. If you will need more time than this to complete your revisions, please reply to this message or contact the journal office at globalpubhealth@plos.org. When you're ready to submit your revision, log on to https://www.editorialmanager.com/pgph/ and select the 'Submissions Needing Revision' folder to locate your manuscript file.

Please include the following items when submitting your revised manuscript:

- A rebuttal letter that responds to each point raised by the editor and reviewer(s). You should upload this letter as a separate file labeled 'Response to Reviewers'.
- A marked-up copy of your manuscript that highlights changes made to the original version. You should upload this as a separate file labeled 'Revised Manuscript with Track Changes'.
- An unmarked version of your revised paper without tracked changes. You should upload this as a separate file labeled 'Manuscript'.

Guidelines for resubmitting your figure files are available below the reviewer comments at the end of this letter.

We look forward to receiving your revised manuscript.

Kind regards,

A/Prof Suman Majumdar

Academic Editor

PLOS Global Public Health

Journal Requirements:

1. We have amended your Competing Interest statement to comply with journal style. We kindly ask that you double check the statement and let us know if anything is incorrect.

2. In the online submission form, you indicated that [Datasets generated or analysed during the project can be made available to The Aurum Institute upon reasonable request.].

All PLOS journals now require all data underlying the findings described in their manuscript to be freely available to other researchers, either

a. In a public repository,

b. Within the manuscript itself, or

c. Uploaded as supplementary information.

This policy applies to all data except where public deposition would breach compliance with the protocol approved by your research ethics board. If your data cannot be made publicly available for ethical or legal reasons (e.g., public availability would compromise patient privacy), please explain your reasons by return email and your exemption request will be escalated to the editor for approval. Your exemption request will be handled independently and will not hold up the peer review process, but will need to be resolved should your manuscript be accepted for publication. One of the Editorial team will then be in touch if there are any issues.

3. If the reviewer comments include a recommendation to cite specific previously published works, please review and evaluate these publications to determine whether they are relevant and should be cited. There is no requirement to cite these works unless the editor has indicated otherwise.

[Note: HTML markup is below. Please do not edit.]

Reviewers' comments:

Reviewer's Responses to Questions

**Comments to the Author**

1. Does this manuscript meet PLOS Global Public Health’s publication criteria?

Reviewer #1: Partly

Reviewer #2: Yes

Reviewer #3: Yes

**********

2. Has the statistical analysis been performed appropriately and rigorously?

Reviewer #1: Yes

Reviewer #2: Yes

Reviewer #3: Yes

**********

3. Have the authors made all data underlying the findings in their manuscript fully available (please refer to the Data Availability Statement at the start of the manuscript PDF file)?

The PLOS Data policy

Reviewer #1: Yes

Reviewer #2: Yes

Reviewer #3: Yes

**********

4. Is the manuscript presented in an intelligible fashion and written in standard English?

Reviewer #1: Yes

Reviewer #2: Yes

Reviewer #3: Yes

**********

Reviewer #1: Overall, good compliance to PLOS publication criteria. Just to minor comments for authors to consider, maybe good to mention what tools (if any) were used for both qualitative and quantitative data management, and were there any ethical considerations for the quantitative data used? Please clarify.

Reviewer #2: 153 How were the CHWs and CHW leaders recruited for focus groups and interviews?

159 Why was data collection so delayed? Is there a risk that the information collected is reflected on CommCare based mHealth app or that there may be recall bias?

- Table 4 is confusing.

The way that active days is defined in the methods (the number of days on which CHWs actually used AitaHealth) suggests that “ÁitaHealth Active days” actually be called AitaHealth Outreach days and actual days should be renamed Active days. The list of indicators says % outreach days used as an outcome (which is the proportion of actual days/outreach days) so I would that language in the table (rather than % active days used)

- Table 6 is also very confusing

Is the Completed household assessments column with n (%) the number of households where this question was completed (and proportion)?

Why is it if the tool is used for TB contact tracing that not 100% of households had a member diagnosed with TB?

- Given the problems with battery life that was mentioned, is there a Is there some way to determine how many days the AitaHealth system actually accessible to use in the field? For example, health worker goes with the tablet but then tablet dies, it may be inaccessible for half the day (or is this what was measured in active days?). This would help strengthen the conclusion that mHealth app’s limitations were primarily due to system constraints rather than user resistance.

468 Was it ever explored why the app was used less on the weekends? This seems to be pointing to a user preference issue rather than a technology issue if there's such marked difference between use on weekdays and weekends.

482 Should the number of entries been a median rather than a mean?

494 94% of households had TB fields completed - is that any entry or each individual logged in the household also had questions asked

590 "CHWs incorporated AitaHealth into their outreach but used it inconsistently, not because of poor design or user resistance but because the conditions needed for sustained engagement were not in place" I don't think that this conclusion can be made without understanding how limiting the technological barriers were

608 What is the impact of the technology on the TB program? Was there a clinical or reporting improvement due to the technology? What was the number or proportion of referrals and did this change after implementation? Was there a change in the timeliness of reporting or the fidelity of the information compared to paper records? Were there more households screened overall?

Was there a change in usage over the timeframe of the study which may have reflected battery life etc?

494 When you say “Over 94% of the 495 2,452 households had TB-related fields completed, including treatment status, 496 symptom screening, and treatment history” do you mean every member of the household had this information filled in or at least one member had this question asked?

Reviewer #3: Overview

This mixed-methods study evaluates AitaHealth implementation for TB contact tracing by community health workers in two South African districts. The manuscript provides valuable insights into real-world mHealth implementation challenges.

However the research questions and sampling strategy need clearer articulation. Additionally, integration between qualitative and quantitative findings could be strengthened throughout. The apparent contradiction between high acceptability and low usage rates requires better explanation. Several outcomes, including device failure rates and safety incidents, lack quantification.

Introduction

● Lines 54-56: The statement "20% were not reached" is ambiguous. Please specify what this gap refers to.

● Lines 69-70: The reference to the Ugandan mHealth trial notes that it "failed to improve outcomes." Please specify which key outcomes the strategy failed to improve

● Lines 60-61: The introduction identifies the challenges of paper-based systems and then introduces mHealth as a potential solution. You could make this link even more explicit by stating directly how mHealth features (e.g., automated reminders, digital data capture) are theorised to address the specific problems of "delays, data loss, and missed follow-ups" that you mention.

● Lines 73-77: The introduction states that the study 'evaluates the tool's acceptability, feasibility, and implementation fidelity' but doesn't present specific research questions. Consider adding explicit research questions e.g. acceptable to whom?

Methods

● The methods don't explain how the two districts were selected or describe the sampling strategy. How did you recruit participants? How did you know when you reached a sufficient sample size?

● The training description is brief. Please provide more detail on the duration of training sessions, whether training was standardised across sites, any competency assessments conducted, and whether refresher training was provided during the implementation period.

● The manuscript mentions that the Ekurhuleni district transitioned from AitaHealth to the CommCare app in 2021 (line 142). Since qualitative data collection in Ekurhuleni continued until May 2022, it is possible that CHWs' experiences with CommCare influenced their reflections on AitaHealth. The authors should acknowledge this in the limitations.

● Line 88: The phrase 'TB has also been noted as the leading cause of disease' is imprecise. Please clarify whether this refers to death, cases or morbidity. And during what year or time period?

● Lines 92-94: The final sentence comparing HIV prevalence between districts is awkward and unclear. Consider revising

● Lines 108-115: While technical specifications are provided for the study tablets, it is unclear whether AitaHealth can be deployed on commercially available devices (e.g., iPads, Samsung tablets) or requires specific hardware

Results

● Table 2 shows the number of participants but lacks demographic information (age, gender, years of experience, education level) that could help contextualise the findings, particularly given comments about older CHWs struggling with technology. Is it possible to include this detail?

● The qualitative quotes lack temporal indicators. Given that data collection spanned different periods (especially in Ekurhuleni from 2019-2022), it would be helpful if you indicated when each quote was collected, as perceptions may have evolved over time.

● Table 5 suggests nearly half of CHWs logged in fewer than 15 times over 9-12 months. This seems inconsistent with claims of acceptability. Could you please explain this? Is the result of the specific barriers mentioned in qualitative findings? Consider adding a section in the results where you integrate the quantitative and qualitative findings.

● The description of hardware and software failures is concerning but lacks quantification. What proportion of devices failed? How many CHWs reverted to paper?

● CHWs expressed significant safety concerns, but it's unclear if any actual incidents occurred. Please clarify whether any devices were stolen or CHWs were harmed

● While data is presented for both sites, there's limited comparative analysis. Given the different contexts (rural vs urban), what differences emerged in implementation experiences between sites?

● There's no assessment of data accuracy. Were any validation checks performed to ensure the quality of entered data compared to paper records?

Discussion

● The opening paragraph summarises findings but there appears to be a contraindication between 'well accepted' and usage on 'fewer than half of outreach days.' In the discussion of fidelity it is suggested that this is driven by system level factors (battery, wifi). Could low usage also reflect rational CHW decisions about when digital tools add value versus create burden?

● While comparing findings to Uganda and Malawi studies, the discussion doesn't address whether these contexts are sufficiently similar for meaningful comparison. Please acknowledge relevant contextual differences.

● The results included perspectives from supervisors and stakeholders, but these voices are largely absent from the discussion. How do supervisor views align or conflict with CHW experiences?

● What can be learned from the high frequency users? Did their success reveal strategies that could inform future implementations?

● The discussion doesn't address long-term sustainability. What happened after the study ended? The mention of Ekurhuleni switching to CommCare suggests AitaHealth wasn't sustained - why?

● The discussion doesn't engage with the economics of mHealth implementation. What are the cost implications of providing devices, support, and training versus potential efficiency gains?

**********

what does this mean?). If published, this will include your full peer review and any attached files.

**Do you want your identity to be public for this peer review?**  If you choose “no”, your identity will remain anonymous but your review may still be made public.

For information about this choice, including consent withdrawal, please see our Privacy Policy

Reviewer #1: No

Reviewer #2: No

Reviewer #3: No

**********

[NOTE: If reviewer comments were submitted as an attachment file, they will be attached to this email and accessible via the submission site. Please log into your account, locate the manuscript record, and check for the action link "View Attachments". If this link does not appear, there are no attachment files.]

While revising your submission, please upload your figure files to the Preflight Analysis and Conversion Engine (PACE) digital diagnostic tool, https://pacev2.apexcovantage.com/. PACE helps ensure that figures meet PLOS requirements. To use PACE, you must first register as a user. Registration is free. Then, login and navigate to the UPLOAD tab, where you will find detailed instructions on how to use the tool. If you encounter any issues or have any questions when using PACE, please email PLOS at figures@plos.org. Please note that Supporting Information files do not need this step.

# Author comment: Author response to Decision Letter 1

**DOI:** 10.1371/journal.pgph.0007120.r002

7 Jan 2026

Attachment Submitted filename: Asibambisane paper rebuttal Letter.docx

# Peer review: Decision Letter 1

**DOI:** 10.1371/journal.pgph.0007120.r003

**Authors:** Ilse Bloom

31 Mar 2026

PGPH-D-25-01506R1

Evaluating the acceptability, feasibility, and implementation fidelity of a multipurpose mobile health (mHealth) app used for Tuberculosis (TB) contact tracing by Ward-Based Outreach Teams (WBOTs) in South Africa

PLOS Global Public Health

Dear Dr. Mudzengi,

Thank you for submitting your manuscript to PLOS Global Public Health. After careful consideration, we feel that it has merit but does not fully meet PLOS Global Public Health’s publication criteria as it currently stands. Therefore, we invite you to submit a revised version of the manuscript that addresses the points raised during the review process.

The manuscript has been further evaluated by three reviewers, and their comments are available below.

Could you please carefully revise the manuscript to address all comments raised by Reviewer 4?

Please submit your revised manuscript by May 15 2026 11:59PM. If you will need more time than this to complete your revisions, please reply to this message or contact the journal office at globalpubhealth@plos.org. When you're ready to submit your revision, log on to https://www.editorialmanager.com/pgph/ and select the 'Submissions Needing Revision' folder to locate your manuscript file.

Please include the following items when submitting your revised manuscript:

- A letter that responds to each point raised by the editor and reviewer(s). You should upload this letter as a separate file labeled 'Response to Reviewers'.
- A marked-up copy of your manuscript that highlights changes made to the original version. You should upload this as a separate file labeled 'Revised Manuscript with Track Changes'.
- An unmarked version of your revised paper without tracked changes. You should upload this as a separate file labeled 'Manuscript'.

Guidelines for resubmitting your figure files are available below the reviewer comments at the end of this letter.

As the corresponding author, your ORCID iD is verified in the submission system and will appear in the published article. PLOS supports the use of ORCID, and we encourage all coauthors to register for an ORCID iD and use it as well. Please encourage your coauthors to verify their ORCID iD within the submission system before final acceptance, as unverified ORCID iDs will not appear in the published article. *Only*  the individual author can complete the verification step; PLOS staff *cannot*  verify ORCID iDs on behalf of authors.

We look forward to receiving your revised manuscript.

Kind regards,

Ilse Bloom

Staff Editor

PLOS Global Public Health

**Journal Requirements:**

If the reviewer comments include a recommendation to cite specific previously published works, please review and evaluate these publications to determine whether they are relevant and should be cited. There is no requirement to cite these works unless the editor has indicated otherwise.

**Additional Editor Comments (if provided):**

[Note: HTML markup is below. Please do not edit.]

Reviewers' comments:

Reviewer's Responses to Questions

**Comments to the Author**

Reviewer #1: All comments have been addressed

Reviewer #2: All comments have been addressed

Reviewer #4: (No Response)

**********

publication criteria?

Reviewer #1: Yes

Reviewer #2: Yes

Reviewer #4: Yes

**********

3. Has the statistical analysis been performed appropriately and rigorously?

Reviewer #1: Yes

Reviewer #2: Yes

Reviewer #4: Yes

**********

4. Have the authors made all data underlying the findings in their manuscript fully available (please refer to the Data Availability Statement at the start of the manuscript PDF file)?

The PLOS Data policy

Reviewer #1: Yes

Reviewer #2: Yes

Reviewer #4: Yes

**********

5. Is the manuscript presented in an intelligible fashion and written in standard English?

Reviewer #1: Yes

Reviewer #2: Yes

Reviewer #4: Yes

**********

**Reviewer #1:**  Good work

**Reviewer #2:**  Thank you for addressing my previous comments. The terminology and tables are clearer.

**Reviewer #4:**  This study evaluated an mHealth application (AitaHealth) for household contact tracing and described the acceptability, feasibility, and implementation fidelity of the mHealth approach. The authors have responded constructively to the first round of reviews. I have several comments and questions for the authors’ consideration.

1. The manuscript would benefit from a clearer description of how AitaHealth was specifically used for TB contact tracing. The tool reads primarily as a general data collection platform.

2. CHWs trained / training completion rate: Listed as a fidelity indicator with "% trained, completion rates" as the intended metric. This is not reported in the quantitative results. Only a narrative description of training is provided.

3. The results remain largely siloed: qualitative findings are presented in one section and quantitative findings in another. Given that this is a mixed-methods study, an integrated results section would strengthen the paper.

4. Did the authors consider including other drivers of app use, such as CHW age, years of service, and training, in the mixed effects model? Could the authors clarify whether these variables were considered for inclusion in the model, and if so, why they were excluded? If the data were not available, this should be acknowledged as a limitation.

5. CHWs used the app on approximately 12% of weekends due to their work commitments, which was acknowledged in the response to reviewers but not clear in the revised manuscript.

6. Each FGD is described as including 12 participants, yet the totals (25 in Ekurhuleni, 43 in uMkhanyakude across two FGDs each) do not consistently add up to 12 per group. This should be clarified.

7. Where possible, the authors may wish to consider including key quantitative findings in the abstract

**********

what does this mean?). If published, this will include your full peer review and any attached files.

**Do you want your identity to be public for this peer review?**  If you choose “no”, your identity will remain anonymous but your review may still be made public.

For information about this choice, including consent withdrawal, please see our Privacy Policy

Reviewer #1: No

Reviewer #2: No

Reviewer #4: No

**********

[NOTE: If reviewer comments were submitted as an attachment file, they will be attached to this email and accessible via the submission site. Please log into your account, locate the manuscript record, and check for the action link "View Attachments". If this link does not appear, there are no attachment files.]

**Figure Resubmissions:**

After uploading your figures to PLOS’s NAAS tool - https://ngplosjournals.pagemajik.ai/artanalysis, NAAS will process the files provided and display the results in the "Uploaded Files" section of the page as the processing is complete. If the uploaded figures meet our requirements (or NAAS is able to fix the files to meet our requirements), the figure will be marked as "fixed" above. If NAAS is unable to fix the files, a red "failed" label will appear above. When NAAS has confirmed that the figure files meet our requirements, please download the file via the download option, and include these NAAS processed figure files when submitting your revised manuscript.

# Author comment: Author response to Decision Letter 2

**DOI:** 10.1371/journal.pgph.0007120.r004

15 May 2026

Attachment Submitted filename: Response to Reviewers 15May26.docx

# Peer review: Decision Letter 2

**DOI:** 10.1371/journal.pgph.0007120.r005

**Authors:** Julia Robinson

30 Jul 2026

Evaluating the acceptability, feasibility, and implementation fidelity of a multipurpose mobile health (mHealth) app used for Tuberculosis (TB) contact tracing by Ward-Based Outreach Teams (WBOTs) in South Africa

PGPH-D-25-01506R2

Dear Mr Mudzengi,

We are pleased to inform you that your manuscript 'Evaluating the acceptability, feasibility, and implementation fidelity of a multipurpose mobile health (mHealth) app used for Tuberculosis (TB) contact tracing by Ward-Based Outreach Teams (WBOTs) in South Africa' has been provisionally accepted for publication in PLOS Global Public Health.

Before your manuscript can be formally accepted you will need to complete some formatting changes, which you will receive in a follow up email. A member of our team will be in touch with a set of requests.

Please note that your manuscript will not be scheduled for publication until you have made the required changes, so a swift response is appreciated.

IMPORTANT: The editorial review process is now complete. PLOS will only permit corrections to spelling, formatting or significant scientific errors from this point onwards. Requests for major changes, or any which affect the scientific understanding of your work, will cause delays to the publication date of your manuscript.

If your institution or institutions have a press office, please notify them about your upcoming paper to help maximize its impact. If they'll be preparing press materials, please inform our press team as soon as possible -- no later than 48 hours after receiving the formal acceptance. Your manuscript will remain under strict press embargo until 2 pm Eastern Time on the date of publication. For more information, please contact globalpubhealth@plos.org.

Thank you again for supporting Open Access publishing; we are looking forward to publishing your work in PLOS Global Public Health.

Best regards,

Julia Robinson

Executive Editor

PLOS Global Public Health

***********************************************************

Reviewer Comments (if any, and for reference):

Reviewer's Responses to Questions

**Comments to the Author**

Reviewer #4: All comments have been addressed

**********

publication criteria?

Reviewer #4: Yes

**********

3. Has the statistical analysis been performed appropriately and rigorously?

Reviewer #4: N/A

**********

4. Have the authors made all data underlying the findings in their manuscript fully available (please refer to the Data Availability Statement at the start of the manuscript PDF file)?

The PLOS Data policy

Reviewer #4: No

**********

5. Is the manuscript presented in an intelligible fashion and written in standard English?

Reviewer #4: Yes

**********

Reviewer #4: My comments have been adequately addressed. Thank you.

**********

what does this mean?). If published, this will include your full peer review and any attached files.

**Do you want your identity to be public for this peer review?**  If you choose “no”, your identity will remain anonymous but your review may still be made public.

For information about this choice, including consent withdrawal, please see our Privacy Policy

Reviewer #4: No

**********
